# Supplementary material for: Interprofessional Skills Learning Guide: A Multimedia E-Book for Small-Group or Individual Learning
Source: MedEdPORTAL. 2016 Jul 8;12:10425. doi: 10.15766/mep_2374-8265.10425 (PMC6464415; doi:10.15766/mep_2374-8265.10425)
Supplement: Supplementary file 1 — A. Interprofessional Skills Learning Guide.epub B. Instructions for Use.docx C. Worksheet.docx D. Interprofessional Skills Learning Guide PDF Version.pdf [file mep-12-10425-s001.zip › _Educational Summary Report_10425.pdf]

MedEdPORTAL®
The Journal of Teaching and Learning Resources

Crossref

Original Publication

OPEN ACCESS

Interprofessional Skills Learning Guide: A Multimedia E-Book for Small-Group or Individual Learning

Heather Ward, MD*, Sharon Elizabeth Card, MD, Dylan Chipperfield, Suzanned M. Sheppard, PhD, Franke Bulk, Wayne Giesbrecht
*Corresponding author: heather.ward@usask.ca

Citation: Ward H, Card SE, Chipperfield D, Sheppard SM, Bulk F, Giesbrecht W. Interprofessional skills learning guide: a multimedia e-book for small-group or individual learning. *MedEdPORTAL*. 2016;12:10425. https://doi.org/10.15766/mep_2374-8265.10425

Copyright: © 2016 Ward et al. This is an open-access article distributed under the terms of the Creative Commons Attribution license.

Appendices

A. Interprofessional Skills Learning Guide.epub
B. Instructions for Use.docx
C. Worksheet.docx
D. Interprofessional Skills Learning Guide PDF Version.pdf

All appendices are peer reviewed as integral parts of the Original Publication.

Abstract

Introduction: Redefining learning space beyond physical classrooms with fixed resources is necessary to address challenges of interprofessional learning in a clinical setting. This multimedia e-book introduces recognized team skills of shared mental models, situational awareness, and the SBAR (situation, background, assessment, and recommendation) communication tool for individual or small-group learning. The e-book was derived from work done to develop an interprofessional small-group interactive learning tool for use in a clinical environment where resources, including meeting space, time, and facilitators, were limited. It is designed for individuals early in their clinical training but who have had previous clinical experience. **Methods:** Utilizing readings, a series of videos, and reflective questions, a virtual narrator guides learners through an interactive case regarding a virtual chronic obstructive pulmonary disease patient preparing for discharge. **Results:** Thirty-two responders evaluated the learning content as being clinically relevant. Comments encouraged all health care providers to become familiar with these interprofessional tools. **Discussion:** Electronic, human, and space resources are often limited, especially in the clinical/education interface of the hospital or clinic environment for embedded interprofessional learning opportunities. The multimedia e-book provides a stand-alone learning resource for individuals or small groups of the same or different professions, with the opportunity for interactive learning with minimal space and human resource requirements.

Keywords

Communication, Small Group, Interactive, Multimedia E-Book, Interprofessional, Collaboration, Team Meeting

Educational Objectives

At the end of this interactive session, participants will be able to:

1. Describe the rationale or importance of interprofessional team skills in health care delivery in the context of their own learning or work experience.
2. Define situational awareness, shared mental models, and the SBAR interprofessional communication tool as team skills and identify examples of each of these team skills demonstrated in the interprofessional discharge planning video.
3. Reflect on or discuss with other group members strategies to implement the team skills described in the e-book in their own learning and practice environments.

Introduction

In Canada, as in many other countries, people are living longer but with more chronic disease.[1-3] Health systems have responded by becoming more specialized within and between professions to address the increasing complexity of health care needs. Patient and family expectations of health care are also evolving, with increased emphasis on coordinated or integrated care and participation in health care decision making.[4] All of these expectations combined result in a necessity for strong interprofessional skills as a requirement for coordinated, safe, and engaged care.[5] Competencies for interprofessional

practice and education are becoming increasingly defined.[5] Three common skills, with their origins in airline safety, are situational awareness, shared mental models, and a shared communication tool such as SBAR (situation, background, assessment, recommendation).[6] The overall goal of team skills is to provide patient-centered care while enhancing individual professional contributions and maximizing available resources.[1] This is increasingly important in providing care for individuals with chronic multisystem disease.

Interprofessional education is defined as a “teaching strategy or educational approach where two or more professionals learn about, with, and from each other to improve collaboration and the quality of care.”[7] The goal of interprofessional education is to engage learners in interactive learning that will enhance their interprofessional collaborative competencies.[7] Interprofessional education is a core component of all health professions education. Challenges of interprofessional education include available content expertise, physical learning space, and information technology resources, especially in a clinical setting. Redefining learning space beyond physical classrooms with fixed resources is a necessary area for development, especially to address challenges of interprofessional learning.[8] Our multimedia e-book is downloadable to the learner’s own electronic device (tablet or laptop) and features a virtual narrator, who leads individual or small-group participants through a case-based interactive learning session of a chronic obstructive pulmonary disease (COPD) patient preparing for acute care discharge. Student-centered learning, either for individuals or small groups of the same or different professions, has the opportunity for interactive learning with minimal space and human resource requirements. Previous clinical experience is necessary as participants are asked to reflect on their own experiences of team-based practice or its absence in a clinical setting.

The multimedia e-book was derived from work done to develop an interprofessional small-group interactive learning tool for use in a clinical environment where resources, including meeting space, time, and facilitators, were limited. Our initial plan was to develop an online learning program with a virtual COPD patient preparing for discharge, utilizing decision trees with clinical information collected through interviews of multiple health care professionals.[9] Logistically, the computer learning program was not sustainable, and an alternative accessible learning tool was sought. Interprofessional skills, situational awareness, shared mental models, and the SBAR communication tool were identified, and written content was added to the clinical information. Our next step was to test the learning content through live sessions with a facilitator and students on clinical rotations from several different professions. Once the content was validated, our final phase was to return to our initial plan of an accessible electronic resource for use in a clinical setting.[10] Media experts at the University of Saskatchewan suggested a multimedia e-book with text and videos that would include a virtual narrator highlighting key learning points and facilitating both individual learning and small-group discussion. Many resources to facilitate case-based interprofessional learning were available in MedEdPORTAL, but a similar, stand-alone e-book was not identified.

Methods

The e-book (Appendix A) is intended for use by individuals with some clinical experience in an acute care setting. The content is an introductory discussion of recognized team skills, shared mental models, situational awareness, and the SBAR communication tool as a framework for approaching complex collaborative practice and their relevance for the provision of safe and expected health care. The clinical scenario, which demonstrates the introduced team skills and provides for a reflective discussion, involves a COPD patient preparing for discharge. The e-book content consists of text reviewing the importance of collaborative practice in our health care system, a brief description of each of the team skills, and reflective questions. The e-book also includes highlighted content from the fictional COPD patient’s chart.

The virtual narrator provides a series of brief video vignettes highlighting the key learning content, guiding the learner through the e-book, and presenting the questions for discussion. The six video vignettes include (1) a description of the links between airline safety and team skills, (2) an in-hospital interview with

the fictional COPD patient, Mr. Sim, highlighting his discharge concerns, (3) the outcome of a not-so-successful discharge, (4) Mr. and Mrs. Sim at home, (5) a successful discharge following an interprofessional team meeting modeling the interprofessional skills, and (6) an interprofessional team meeting.

Reflective questions are included throughout the e-book to facilitate learning. The final chapter collates all of the reflective questions as well as giving links to the relevant videos, providing a structure for small-group discussion. A worksheet (Appendix C) is provided to assess the learners' engagement with the material and use of the reflection questions.

The e-book is intended for trainees in all health care professions who have some clinical experience. It is best suited to individuals in early clinical training. This would include senior medical students or senior undergraduate trainees in all health professions who have participated in a clinical placement. The clinical experience does not need to be extensive but should be sufficient enough that the learner will be able to reflect on his/her own experience of working in teams and potential application of the team skills in a practice environment. The book is designed for individual reflective or small-group discussion for learning.

The e-book is a stand-alone learning tool with no supplementary resources necessary. The e-book is provided in the .epub format and can be downloaded to tablets, laptops, or desktop computers. The majority of trainees have their own laptop or tablet that they carry with them in clinical settings. In our experience, learners have used their own laptops or tablets to download and review the e-book. If individual laptops or tablets are not available, the e-book can be downloaded onto a single computer and projected onto a screen for the small group to review and discuss content. Projecting the e-book would require a facilitator to arrange equipment and coordinate pace to allow each individual sufficient time to review the content. A user's guide (Appendix B) is provided to help troubleshoot technical issues. A PDF version (Appendix D) is provided if the users are unable to resolve compatibility issues with the e-book version.

For individual learning, it takes approximately 60 minutes to complete the readings, review the videos, and reflect on the defined questions. For small-group learning, 90 minutes total should be allotted. Sixty minutes should be allotted for independent review of the e-book, either individually or in pairs, and 30 minutes for the group learning activities and discussion that are colocated in the final chapter. For small-group learning, individuals can be asked to download and review the content of the e-book on their own prior to a designated meeting time and place. Approximately 30 minutes should be allowed for the e-book download. The discussion questions as well as links to the relevant videos are presented in the final chapter.

Small groups will benefit from having a facilitator who is familiar with the e-book content and may be able to troubleshoot basic electronic challenges and coordinate discussion. This individual does not need to be a content expert on collaboration. A virtual narrator, in a series of video vignettes, guides the learners through the e-book content. With the use of individual tablets or laptops, any room of sufficient size for the group should also ideally have Wi-Fi access if the learners will be downloading the e-book during the session. No other resources are required.

Results

The initial feedback on clinical and learning content was evaluated from 32 responses (26 internal medicine postgraduate trainees and six physiotherapy students) using a 3-point rating scale. The feedback demonstrated that in a clinical environment in the context of a COPD patient preparing for discharge, shared mental models were 66% very useful and 34% somewhat useful, situational awareness was 75% very useful and 25% somewhat useful, and SBAR was 75% very useful, 22% somewhat useful, and 3% not useful.[9] Comments encouraged all health care providers to become familiar with these tools. At a recent international medical graduate orientation, nine trainees rated the e-book as an 8.9 out of 10 for useful

information. Written comments included the following: “the session was simplistic and yet informative,” “the video examples were great,” “useful e-book with embedded videos,” and “interactive nature of the session; e-book and discussing cases (helped me to learn).” Our initial evaluation, although not identifying learning outcomes, acknowledges the method of content delivery (the e-book) as being accessible and acceptable for use.

Discussion

A realist review of Internet-based education identifies two key findings that align with two theories supporting online learning.[11] The first finding, Davis’ technology acceptance model, is associated with actual use of an Internet-based learning technology, including perceived usefulness, ease of use, and compatibility with learning needs.[11] The second finding, Laurillard’s model of interactive dialogue, is associated with interactivity as an effective component of Internet learning technology.[11] Learners want to interact with a tutor, including a virtual tutor, or with other group members. Although the number of individuals providing feedback was small, there was overwhelming agreement that the multimedia e-book was informative and accessible. Methods of interaction include both the virtual narrator, who provides the content expertise and questions to facilitate group discussion, and the concluding chapter, which links all of the learning resources and associated discussion questions into one section. Clinical experience, but not content expertise, is the only requirement to facilitate discussion within the context of the e-book. Initial evaluation demonstrates that the multimedia e-book meets the essential requirements (usefulness and interactivity) of an effective Internet-based learning resource.

The advantage of an e-book is that it does not need regular maintenance. There are, however, challenges. The main one is the need to download an e-reader program for Windows and Android devices that will support .epub documents. Currently, there is no embedded reader program that will display multimedia content of both text and videos in these operating systems. This two-step process of a software download and the e-book download may add 15 to 30 minutes as background time. There is the potential for limitation in accessibility, depending on local technical security settings for program downloads, specifically, Adobe Digital Editions. Assistance from local technology support maybe required. Apple products, such as Macbooks and iPads, provide a native e-reader and thus the most seamless viewing of the e-book.

In small-group sessions, if time is allowed for everyone to review the e-book content, there is a different pace at which individuals or pairs review the material. A designated group leader is helpful in pacing the group, including allotted time to review the material and then to pace the discussion through each of the learning activities and reflective questions. Expertise is not required by the designated group leader; however, it would be beneficial if the leader has reviewed the content beforehand to be familiar with the reflective activities and essential videos.

Overall, the e-book provides the opportunity for a flexible learning resource with minimal fixed space and human resource requirements for introducing the team skills of situational awareness, shared mental models, and the SBAR communication tool in an interprofessional clinical setting. We will continue to evaluate the e-book for ease of use and time requirements necessary for small-group discussion.

Heather Ward, MD: Associate Professor, Department of Medicine, University of Saskatchewan College of Medicine

Sharon Elizabeth Card, MD: Associate Professor, Department of Medicine, University of Saskatchewan College of Medicine

Dylan Chipperfield: Manager, LiveWell Chronic Disease Program, Saskatoon Health Region

Suzanned M. Sheppard, PhD: Director of Interprofessional Practice, Education, and Research, Saskatoon Health Region

Franke Bulk: Manager of E-learning and Innovative Programs, Gwenna Moss Centre for Teaching Effectiveness, University of Saskatchewan

Wayne Giesbrecht: Senior Media Developer, University of Saskatchewan

Disclosures
None to report.

Funding/Support
Saskatchewan Academic Health Sciences Network.

Ethical Approval
Reported as not applicable.

References

1. Hall P, Weaver L. Interdisciplinary education and teamwork: a long and winding road. *Med Educ.* 2001;35(9):867-875. http://dx.doi.org/10.1046/j.1365-2923.2001.00919.x
2. *Global Status Report on Noncommunicable Diseases 2014*. Geneva, Switzerland: World Health Organization; 2014.
3. Vedel I, Monette M, Beland F, Monette J, Bergman H. Ten years of integrated care: backwards and forwards. The case of the province of Québec, Canada. *Int J Integr Care.* 2011;11(5):e004. http://dx.doi.org/10.5334/ijic.574
4. Novelli WD, Halvorson GC, Santa J. Recognizing an opinion: findings from the IOM Evidence Communication Innovation Collaborative. *JAMA.* 2012;308(15):1531-1532. http://dx.doi.org/10.1001/jama.2012.13369
5. National Interprofessional Competency Framework. Canadian Interprofessional Health Collaborative Web site. http://www.cihc.ca/files/CIHC_IPCompetenciesShort_Feb1210.pdf. Accessed January 7, 2016.
6. Carbo AR, Tess AV, Roy C, Weingart SN. Developing a high-performance team training framework for internal medicine residents: the ABC's of teamwork. *J Patient Saf.* 2011;7(2):72-76. http://dx.doi.org/10.1097/PTS.0b013e31820dbe02
7. Defining IPE. Centre for the Advancement of Interprofessional Education Web site. http://caipe.org.uk/resources/defining-ipe/. Accessed January 7, 2016.
8. Nordquist J, Laing A. Spaces for learning—a neglected area in curriculum change and strategic educational leadership *Med Teach.* 2014;36(7):555-556. http://dx.doi.org/10.3109/0142159X.2014.917288
9. Ward H, Card S, Chipperfield D, Sheppard MS. Simulated team scenario: a pilot case-based virtual learning tool for interprofessional education and practice. Presented at: Secrets of Success; August 30, 2011; Vienna, Austria.
10. Ward H, Sheppard S, Chipperfield D, Card S. Simulated learning by clinical trainees of communications strategies: facilitating development of collaborative competency in an acute care environment. Poster presented at: Association of Medical Educators of Europe Meeting; August 24-28, 2013; Prague, Czech Republic.
11. Wong G, Greenhalgh T, Pawson R. Internet-based medical education: a realist review of what works, for whom and in what circumstances. *BMC Med Educ.* 2010;10:12. http://dx.doi.org/10.1186/1472-6920-10-12

Received: October 8, 2015 | **Accepted:** April 28, 2016 | **Published:** July 8, 2016
