# Supplementary material for: Interprofessional Skills Learning Guide: A Multimedia E-Book for Small-Group or Individual Learning
Source: MedEdPORTAL. 2016 Jul 8;12:10425. doi: 10.15766/mep_2374-8265.10425 (PMC6464415; doi:10.15766/mep_2374-8265.10425)
Supplement: Supplementary file 1 — A. Interprofessional Skills Learning Guide.epub B. Instructions for Use.docx C. Worksheet.docx D. Interprofessional Skills Learning Guide PDF Version.pdf [file mep-12-10425-s001.zip › B. Instructions for Use.docx]

**Instructions for Use**

*Instructions for Learners:*

The eBook consists of an introductory section followed by 7 short chapters each containing text, videos, and video vignettes of a virtual narrator who will guide you through the learning content. Chapters 1 and 2 introduce the interprofessional team skills of situational awareness, shared mental models, and SBAR (situation, background, assessment, recommendation) communication tool. These skills are applied to a hospital patient with COPD, Mr. Sim, preparing for discharge followed by his concerns of a not so good discharge, the video content in chapter 3. Chapter 4 contains a video of an interprofessional team meeting discussing Mr. Sim’s discharge using the interprofessional team skills. Chapter 5 is Mr. Sim in the community following his interprofessional discharge and chapter 6 concludes or summarizes the learning content of the eBook. Chapter 7 collates the videos and tasks for small group discussion. There are 5 tasks you will be asked to complete individually as you reflect on the content of the eBook and your own clinical experience. Please complete each of the tasks as outlined on the attached work sheet.

The eBook can be used for individual learning or group learning. If you are participating in individual learning, please complete the attached work sheet. If you are participating in small group discussion, please complete the eBook work sheet on your own and bring the completed work sheet to the small group discussion. It will take approximately 60 minutes to review the eBook content on your own and 30 minutes to discuss as a group.

*Instructions for Coordinators/Facilitators*

The eBook is a stand-alone educational resource. There is a virtual narrator, embedded as a series of video vignettes, who will guide the learner through the contents of the eBook and review how to approach assigned tasks. Each small group participant should review the eBook and complete the work sheet before attending the small group. Sixty minutes is recommended for individual review and completion of the work sheet and 30 minutes for small group discussion.

You are not required to be a content expert. Your interprofessional clinical experience that you bring to the small group discussion will be beneficial for the group learning. Instructions for downloading the eBook and the recommended readers are provided in the section below. Your IT department may need to assist in downloading the eReader program (Adobe Digital Editions) depending on the security settings of your institution computer systems. Please review and complete the same worksheet as the students to familiarize yourself with the material and tasks. There are no ‘right’ answers’ to any of the tasks; instead, they are meant to be an opportunity to build on past clinical experiences and bring new ideas to future interprofessional clinical practice.

How to assess learning? The eBook content is an introduction to the interprofessional team skills previously outlined. Assessment of learning includes being able to define and discuss examples of each of these skills either from the interprofessional team video in the eBook or from real life clinical experience. The student should include rationale in their discussion as to why interprofessional team skills are necessary in today’s health care system in order to meet needs of individuals with multisystem chronic disease and often complex social settings. The attached work sheet for completing each task provides you with an opportunity to review the student’s learning.

*EBook details and required eReaders*

The eBook is published as a ‘flowable’ document allowing for font size and spacing to be individually adjusted.  With a flowable document, page numbers will be different, depending on chosen font size, and there may be blank pages as the eReader reformats the book to adjust for individual preferences.  The Table of Contents and sub-headings, as well as the videos, will provide a structure for identifying location in the eBook for shared references.  The final chapter collates links for the learning videos as well as the assigned reflective tasks.

An eReader that supports ePub 3 documents is required to view the eBook because of the embedded videos.  Please use the readers recommended below.  For some users, facility IT permission may be required to download and/or install pub reading software.

Mac laptop or iPad:

The Apple iBooks reader

 Android and Windows (as well as Mac)

[http://www.adobe.com/solutions/ebook/digital-editions/download.html](http://www.adobe.com/solutions/ebook/digital-editions/download.html)

After downloading Adobe digital editions,

click ‘File’

open ‘New’ and save the Ebook through the link provided in MedEdPortal

Two alternative links to the eBook content are provided below.  Please note, the eBook content for these two links has not been peer reviewed by MedEdPortal.

[https://itunes.apple.com/ca/book/interprofessional-skills-learning/id960802721?mt=11](https://itunes.apple.com/ca/book/interprofessional-skills-learning/id960802721?mt=11)

Link for Android and Window users:

[https://books.google.ca/books?id=N2hgBgAAQBAJ&lpg=PP1&pg=PP1#v=onepage&q&f=false](https://books.google.ca/books?id=N2hgBgAAQBAJ&lpg=PP1&pg=PP1#v=onepage&q&f=false)
