# Supplementary material for: Interprofessional Skills Learning Guide: A Multimedia E-Book for Small-Group or Individual Learning
Source: MedEdPORTAL. 2016 Jul 8;12:10425. doi: 10.15766/mep_2374-8265.10425 (PMC6464415; doi:10.15766/mep_2374-8265.10425)
Supplement: Supplementary file 1 — A. Interprofessional Skills Learning Guide.epub B. Instructions for Use.docx C. Worksheet.docx D. Interprofessional Skills Learning Guide PDF Version.pdf [file mep-12-10425-s001.zip › C. Worksheet.docx]

**Work Sheet**

1. eBook outline

A. Introduction: to the virtual narrator and eBook content

B. Chapter 1:Interprofessional team skills and the provision of care for multisystem chronic disease.

Video: Crew Resource Management in the airline industry

C. Chapter 2: an introduction to Situational Awareness, Shared Mental Models and SBAR communication tool as skills to improve interprofessional teamwork.

D. Chapter 3: Clinical Information: a not so good discharge

Video: Mr. Sim discusses his concerns about discharge from hospital

Task 1: identify Mr. Sim’s care goals and the health care interprofessional team

Video: A not so good discharge

Task 2: reflect on your experience with hospital discharges

E. Chapter 4: Interprofessional discharge planning: applied use of situational awareness, shared mental models and SBAR

Video: An interprofessional team meeting planning Mr. Sim’s discharge

Task 3: identify examples of situational awareness, shared mental models, and SBAR from the video.

Task 4: Practicing SBAR

F. Chapter 5: Mr. Sim in the community – outcome of team skills

Video: Mr. Sim

G. Chapter 6: Conclusion

Task 5: Taking these team skills back to your practice or learning environment

H. Chapter 7: small group discussion: summary of assigned tasks and links to the learning videos.

**Task 1: Identify Mr. Sim`s care goals and the healthcare professional team**.

| **Discharge Concerns** | **Healthcare Professional(s)** |
| --- | --- |
|  |  |
|  |  |
|  |  |
|  |  |
|  |  |

**Task 2:**

**Discuss positive and negative discharge experiences you have observed in clinical experience.**

This may include hospital discharges or transitions to other care environments. As you reflect on your experience with transitions of care, describe the rationale or importance of interprofessional team skills in health care delivery.

**Task 3: Identify examples of situational awareness, shared mental models and SBAR from the interprofessional team video (Chapter 4).**

| Team Skill | Definition | Example |
| --- | --- | --- |
| Situational Awareness |  |  |
| Shared Mental Model |  |  |
| SBAR (situation, background, assessment, recommendation) |  |  |

**Task 4: Practicing SBAR**

Select one of Mr. Sim`s care goals you identified in task 1 and write out an SBAR presentation of this care goal for the team meeting.

Care goal:

| Situation |  |
| --- | --- |
| Background |  |
| Assessment |  |
| Recommendation |  |

**Task 5: taking these team skills back to your practice or learning environment**. Reflect or discuss strategies to implement these interprofessional team skills to better meet healthcare needs of patients and families.
