# Supplementary material for: Interprofessional Skills Learning Guide: A Multimedia E-Book for Small-Group or Individual Learning
Source: MedEdPORTAL. 2016 Jul 8;12:10425. doi: 10.15766/mep_2374-8265.10425 (PMC6464415; doi:10.15766/mep_2374-8265.10425)
Supplement: Supplementary file 1 — A. Interprofessional Skills Learning Guide.epub B. Instructions for Use.docx C. Worksheet.docx D. Interprofessional Skills Learning Guide PDF Version.pdf [file mep-12-10425-s001.zip › D. Interprofessional Skills Learning Guide PDF Version.pdf]

Interprofessional Skills Learning Guide

Virtually Narrated Small Group Discussion

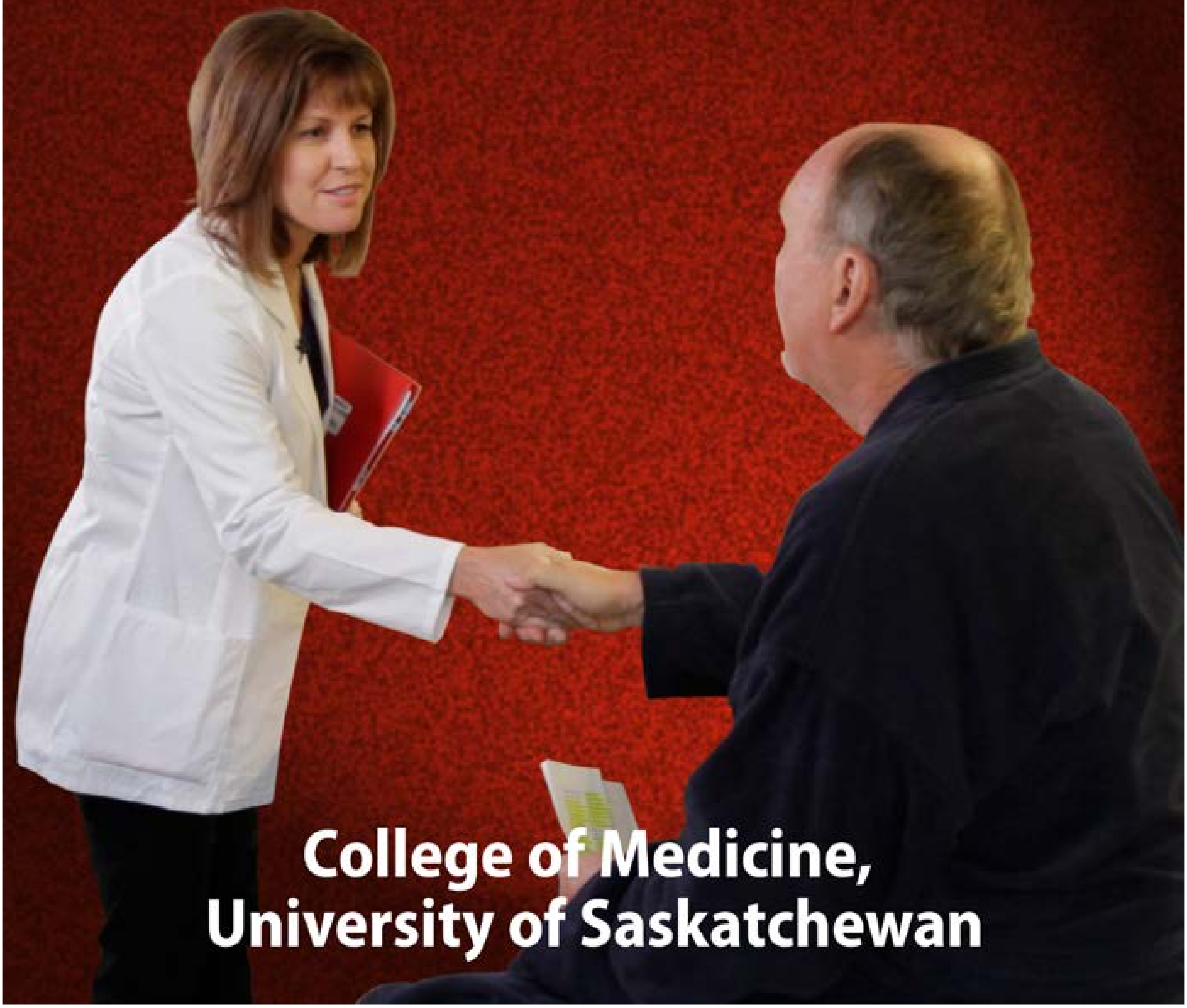

College of Medicine,
University of Saskatchewan

Interprofessional skills learning guide:

Virtually narrated discussion of Situational Awareness, Shared Mental Models, and SBAR communication tool in an acute care general medicine unit.

Authors:

Heather Ward, MD MSc FRCPC, University of Saskatchewan

Dylan Chipperfield, BSc. Kin, University of Saskatchewan

M. S. Sheppard, BSc. (Eng), MSc, BScPT, PhD, Saskatoon Health Region

Frank Bulk, M.Ed., University of Saskatchewan

Sharon E. Card M.D. MSc, FRCPC, University of Saskatchewan

Video Cast

Thanks to our cast of actors and professionals who participated in the video segments of this ebook.

Max Hansen: Mr Sim

Janet Ingram: Mrs. Sim

The Interprofessional Team:

Alex Nelson, BSc, MD

Vicky Kennedy, RN, BN, MN, CRE

Madeleine Cameron, RD CNSC

Jeff Sturgess, BA BSW RSW

Justin Kosar, BSc. BSP

Introduction: The content and learning opportunities of this interprofessional ebook

Welcome to the interprofessional skills ebook. Anyone with acute care clinical experience, both practicing health care professionals and clinical trainees, is invited to participate in the content and activities of this ebook in order to learn more about interprofessional team skills. Learning will be case-based as we discuss a not so successful followed by a successful hospital discharge. The interprofessional team skills discussed are situational awareness, shared mental models and the SBAR communication tool.

Introduction to the virtual narrator and the ebook

This ebook is divided into two sections. The first section is for individual learning and the second section is for small group discussion. Contents of the ebook can be reviewed independently with the opportunity to become familiar with the interprofessional team skills. As an independent learner, you will be reading, watching videos, and reflecting on your own clinical experience in chapters 1 through 6. The second section, chapter 7, is for small group discussion. The value of small group discussion is to learn from each other's experiences and to be able to practice the team communication skill SBAR. Prior to participating in the group discussion, each individual must first complete the independent learning section (chapter 1 to 6) with the intent to bring your reflections and case based problem list to the group discussion. Groups of 3 to 6 participants with representation from more than one healthcare profession is ideal, but discussion by smaller groups or those from the same profession will also provide learning opportunities for team skills. It is estimated it will take 45 to 60 minutes to complete the individual learning content and tasks and an additional 30 to 45 minutes to work through the learning tasks as a small group.

As a brief outline to the ebook, Chapters 1 and 2 provide background information on the need for interprofessional team care in current and future healthcare delivery. The interprofessional team skills, and their background in the airline industry, will then be introduced. In chapter 3 we will meet Mr. and Mrs. Sim, a virtual COPD patient and his wife, who provide the clinical information and context to be used for your own reflective learning and for group discussion. Chapters 4, 5 and 6 will facilitate reflection on interprofessional team skills and their application to the practice environment. Although resources for our virtual COPD patient from our health region are discussed, these same team skills can be applied in multiple different clinical settings utilizing your own available health care resources. In the final chapter, you will be asked the same reflection questions you were as an individual learner, but will be asked to discuss your thoughts with the group. Links to previous videos, especially for the clinical information will be provided so you can refer back to any previously covered information needed for your group discussion.

Let's begin by meeting our virtual narrator, Dylan. He will introduce key content, present the three team skills discussed in this ebook and guide you through the group learning activities. Reflecting on previous clinical experiences and discussing these experiences will optimize your learning. Throughout the ebook, time will be designated for reflection on past experiences allowing you to explore these concepts.

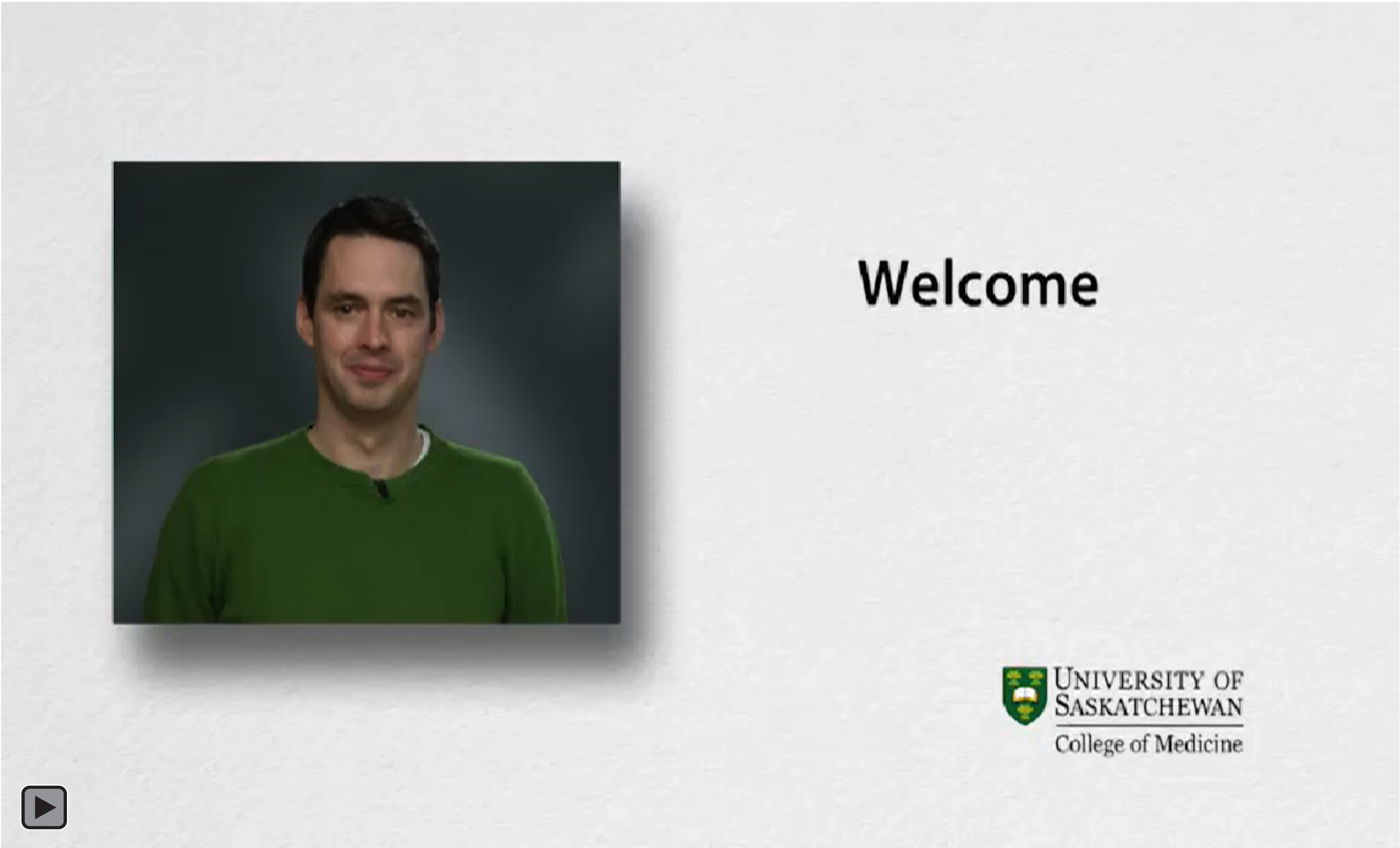

Introducing Mr. Sim: our virtual COPD patient preparing for hospital discharge

This is Mr. Sim our simulated patient. He has COPD, is recovering in hospital from a recent COPD exacerbation and has several concerns as he prepares for discharge home.

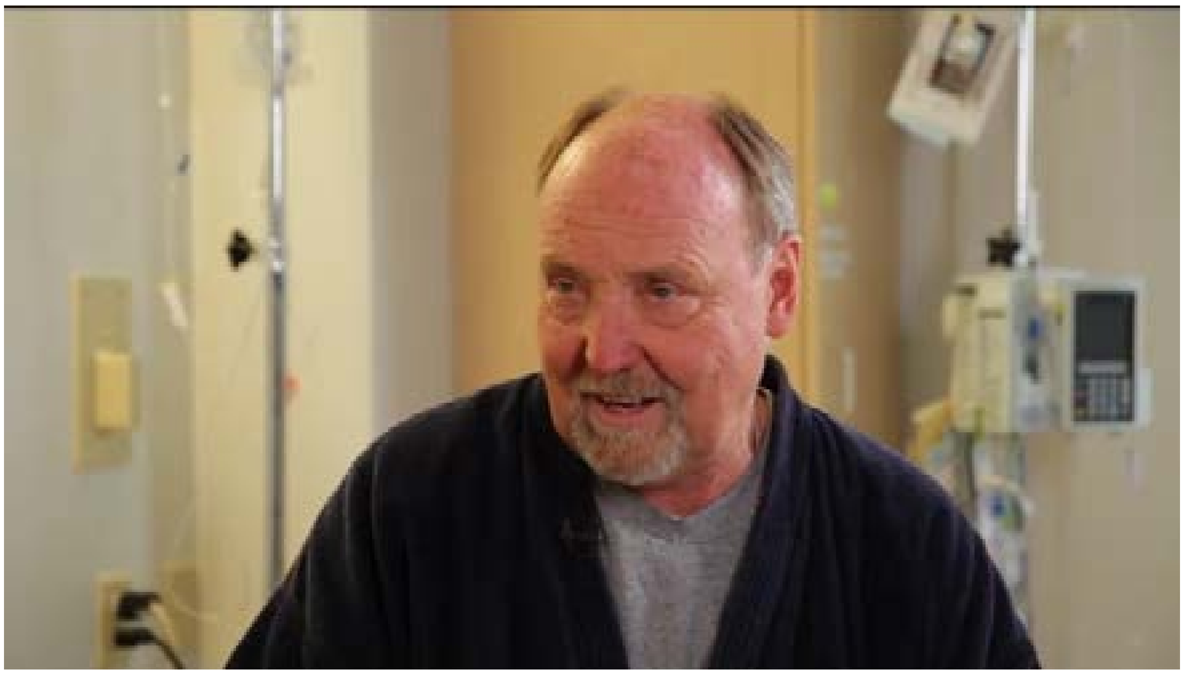

Clinical details, including his hospital chart and an interview with one of his healthcare providers are provided in chapter 3. As with the acute care patients we commonly see, Mr. Sim's discharge concerns are both medical and social. He and his wife need to identify their needs as they make the transition from hospital to home. In chapter 3 Mr. and Mrs. Sim review his not so effective transition to home. In chapter 4, an inter professional team of health care providers applies the team skills to plan a discharge to address Mr. Sim's care goals.

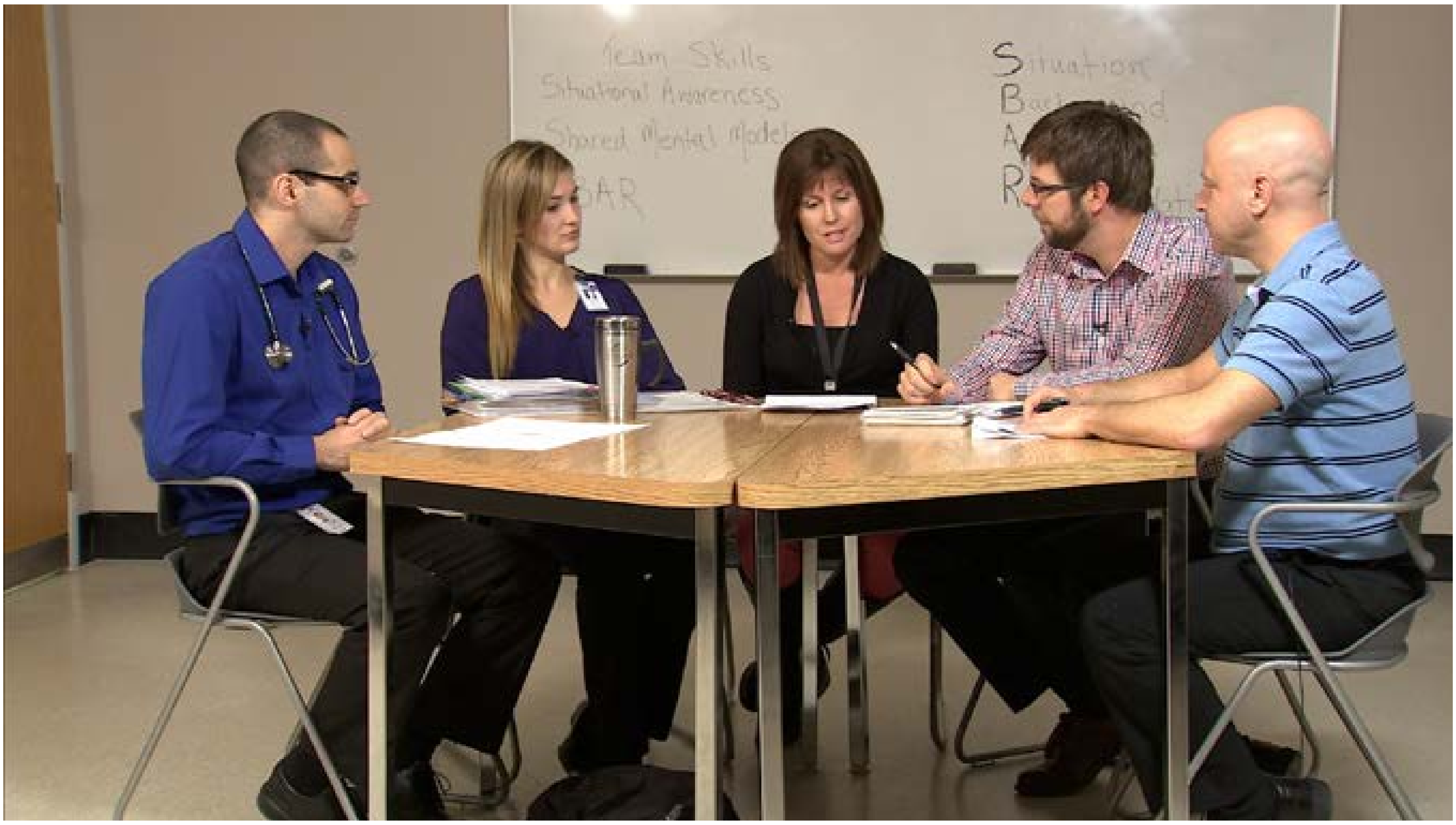

Before presenting the detailed clinical information and discharge planning, you will have opportunity to review background information on the prevalence of multi system chronic diseases and their influence on the direction of health service delivery and the need for inter professional team care.

Chapter 1: Interprofessional Team Skills: Fundamental for current and future healthcare

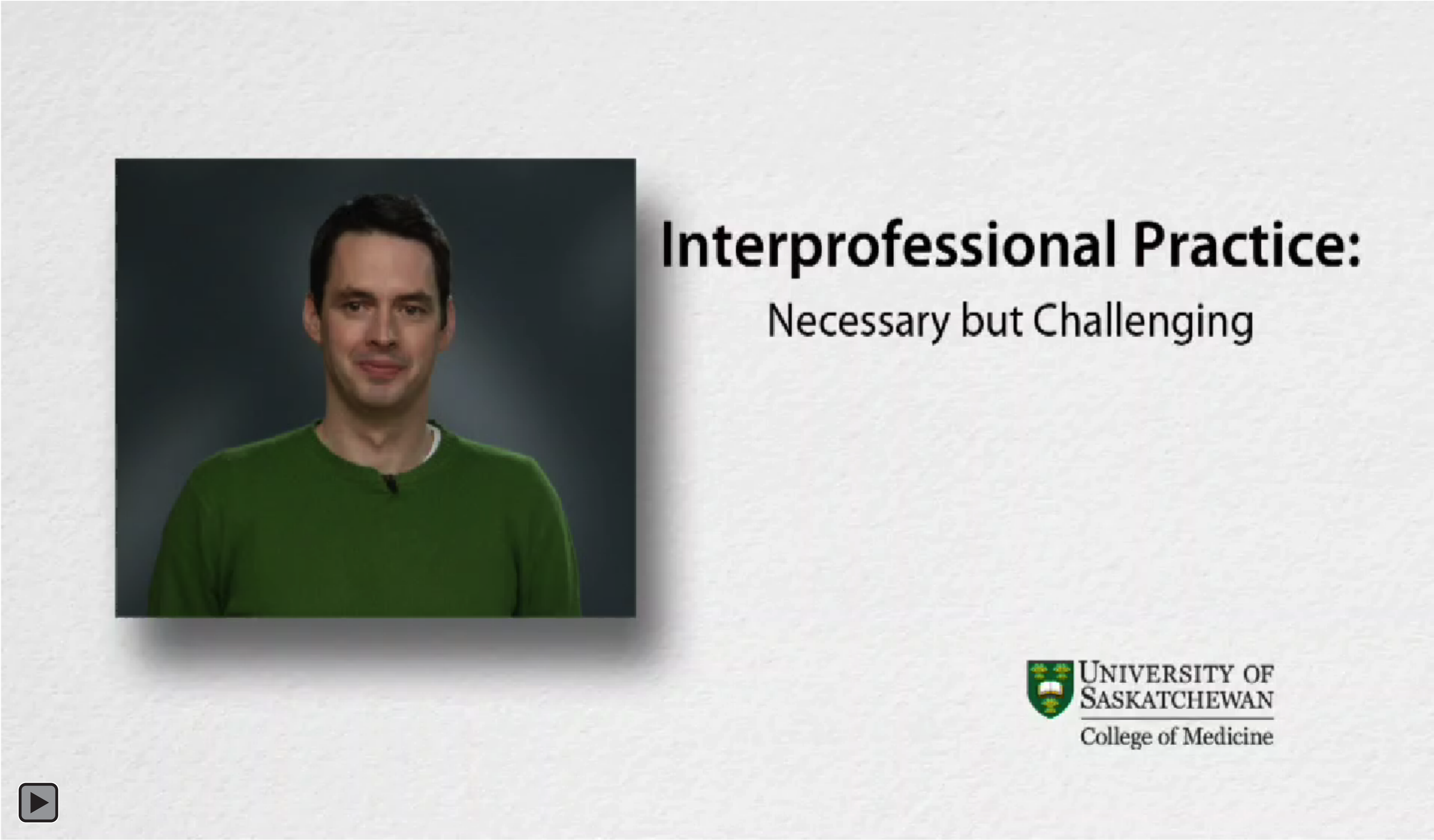

Interprofessional practice: important for and challenging to providing patient care.

Chronic diseases currently are the leading cause of mortality in the world representing 63% of all deaths, according to the World Health Organization. Management of chronic disease requires interprofessional care. As a result, teamwork is and will become an increasingly important component of healthcare delivery (Schmutz 2013).

"Chronic diseases are currently the global leading cause of mortality." (World Health Organization – WHO, 2010)

Although having the knowledge and skills provided by individual healthcare professions is necessary for good patient care, possessing interprofessional team skills is equally important in order for all healthcare professionals to effectively work together for comprehensive care.

Reflecting on your experiences as a healthcare provider, who are the patients you most commonly see in hospital? Is their care, both medical and social, always met? What are the challenges to team care in hospital? How do you think better healthcare could be provided to the patients you most commonly see?

Why Team Skills are Necessary for Healthcare of multi system chronic disease

There are a number of reasons why team skills are necessary for healthcare professionals. Let's review both the rationale, and challenges of interprofessional care.

1. **Providing care for individuals with multiple chronic medical conditions is a key driver for health service delivery changes.**

   Chronic diseases are the leading cause of mortality in the world. In Canada, as in many other countries, people are living longer and in this setting individuals acquire more than one chronic disease in addition to the physiologic changes of aging. As a result, Hall (2001) describes the focus of health care delivery as evolving from a 'model of cure' to a 'model of caring' for chronic disease including controlling symptoms, optimizing level of functioning and quality of life. In addition to the demographics, expectations and needs of people accessing healthcare are also changing. Inpatient surveys of people with chronic disease, indicate that healthcare needs are not always effectively and efficiently met (Novelli, Parke). Social, cultural and emotional needs must also be addressed in addition to medical needs for comprehensive patient-centred care

2. **Providing safe interprofessional care that engages patients and family as team members is a complex task.**

   Although the majority of patients who seek healthcare have multiple chronic diseases in the context of other personal needs, health service delivery remains focused on disease. Often this is on a single disease. There are COPD or congestive heart failure clinics, for example, each providing specialized care for their individual disease process. For the patient, the result is often conflicting recommendations for therapy with no coordinated approach to care and care that they may not be able to access within individual contexts. Multiple authors identify that the full spectrum of patient care needs are not consistently met as either overlap or gaps in care occur and patient safety is compromised (Parke, Vedel, Wright). Expectations are also not being met. Novelli found that only 54% of patients with chronic illnesses report that they are receiving coordinated care they would like to have. In the context of long term management of chronic diseases, patients and families are key team members as ownership and provision of care goals is under their direction, with engagement by the healthcare professional team.

3. **Professional and individual disease focused silos prohibit coordinated interprofessional care**

   Each health care profession brings its own unique professional knowledge and skills to patient care. Subsequently, each health care professional, when looking at the same situation, will identify different issues. It is these unique or multifaceted professional perspectives that provide well established challenges to providing collaborative or team care.

   Meeting patient personal goals of care, or providing patient-centred care, requires the expertise of several different healthcare professionals. Silos of disease-focused healthcare and of specialized professional practice challenge teamwork.

4. **Communication is key to facilitate comprehensive collaborative care to bridge these silos.**

   Communication is challenged by the complexities of multiple medical problems and individual professional expertise that needs to be communicated within and between professions and with patients and families. Hall describes how common or familiar terminology of each profession simplifies communication within each profession, but is a barrier to communication between professions. In addition, a traditional hierarchy between professions and the educational hierarchy between clinical trainees and supervisors limits communication (Hall 2005). Clear and relevant communication of health care providers' roles for each individual patient is required for safe care that addresses self-identified patient needs.

5. **Team skills: Situational awareness, shared mental models and SBAR communication tool, are necessary for safe, comprehensive and satisfactory patient care.**

   The changing landscape of expectations on healthcare systems is resulting in the transition of healthcare service delivery to interprofessional or team-based care that must address a broad spectrum of medical and social issues. Achieving team function in the setting of a fast paced hospital environment is challenging. Other work environments where quality team work is essential for both high level performance and safety, as in the airline industry, have applied situational awareness, shared mental models, and the SBAR communication tool with effective results. Absence of team skills can result in poor outcomes.

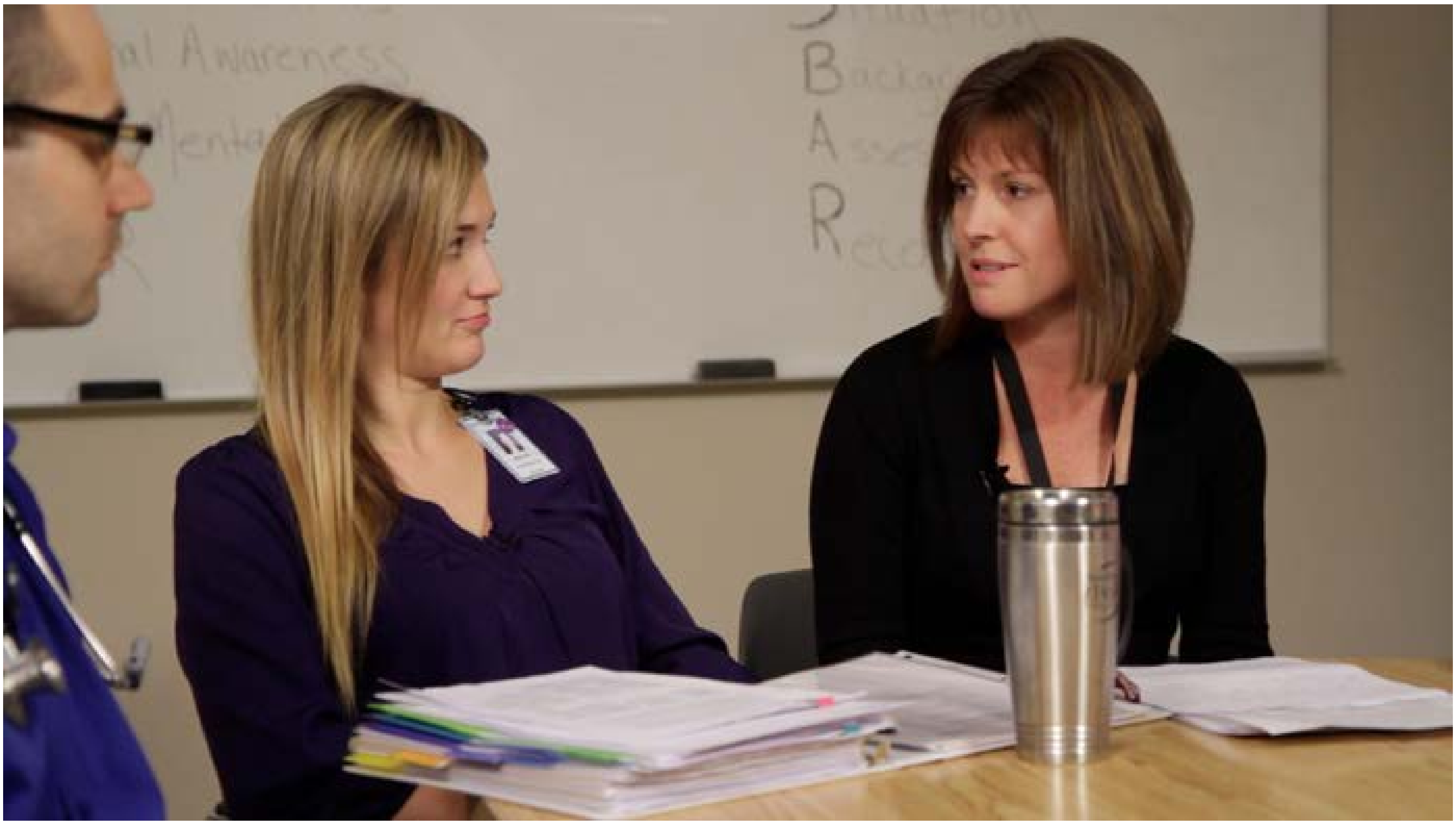

Three frequently applied team skills for interprofessional care are:

- **Situational Awareness**: being aware of what is happening around you
- **Shared Mental Models**: common goals and processes
- **SBAR (situation, background, assessment, recommendation)**: a communication technique that facilitates the necessary communication for participation by all team members in order to achieve patent care goals.

Teamwork and the Airline Industry (Crew Resource Management)

In this section we review the history of crew resource management and lessons learned from the airline industry, the source of our teamwork skills.

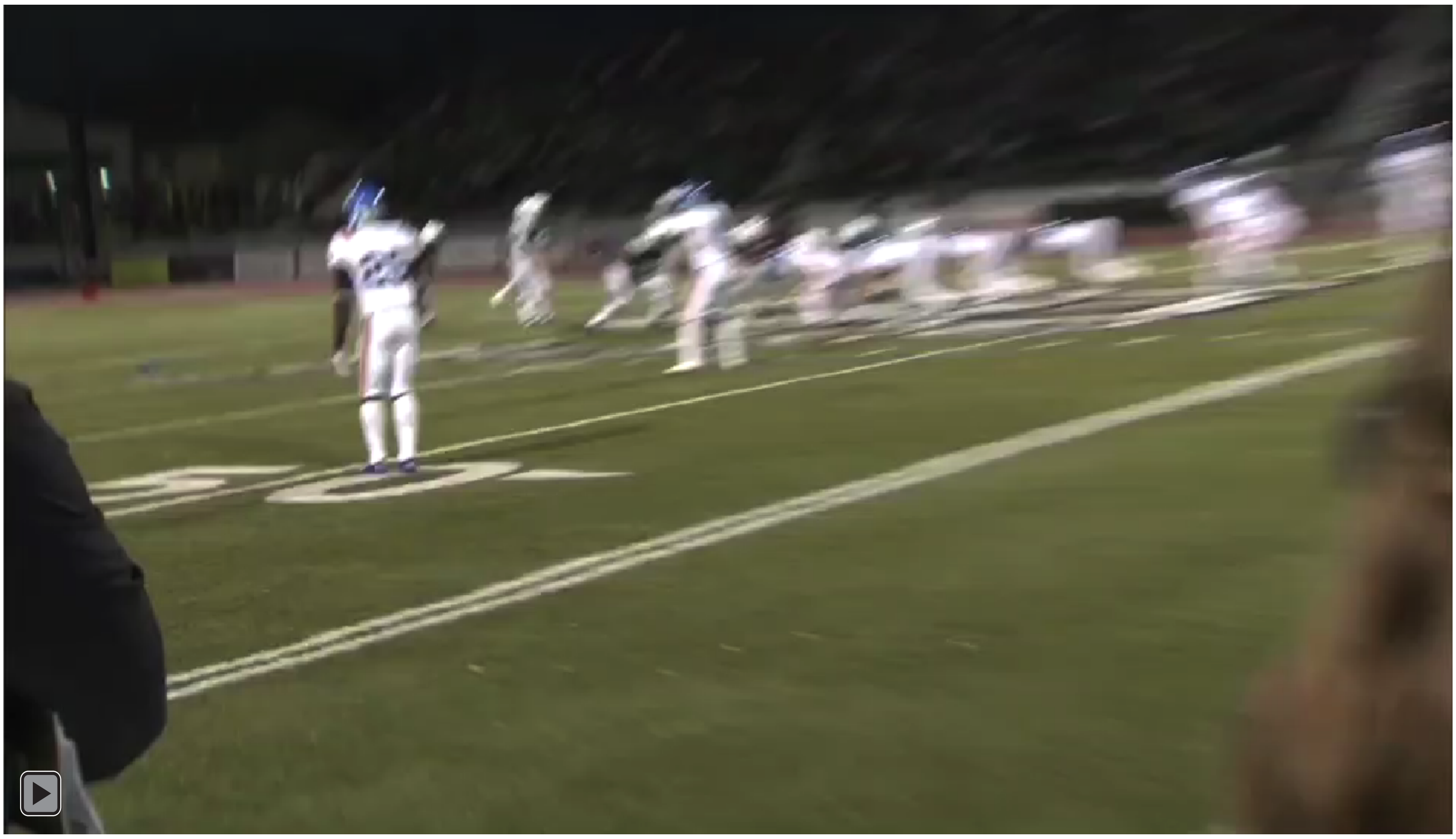

Video ©2015 University of Saskatchewan. Stock footage licenced from Videoblocks, www.videoblocks.com

As a result of the 1972 incident, the airline industry developed and implemented tools for teamwork that have proven to be effective in emergency situations.

These same skills as used in the airline industry, including: **Situational Awareness**, **Shared Mental Models** and **SBAR**, have been introduced into a variety of clinical settings, including care of patients with multiple chronic diseases.

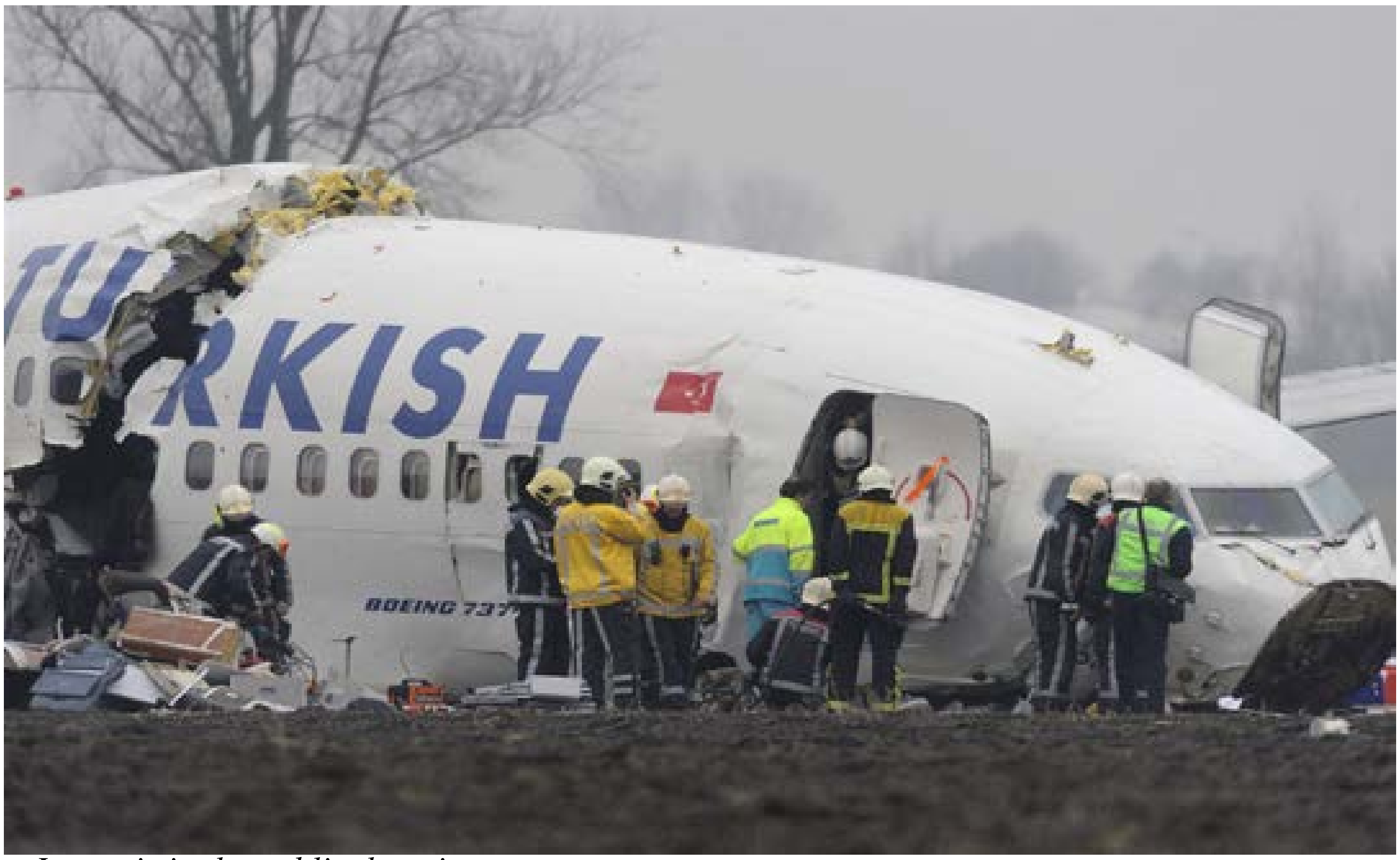

Image is in the public domain:
https://flic.kr/p/63n6aT

Chapter 2: Situational Awareness, Shared Mental Models, And SBAR: tools to improve interprofessional team work

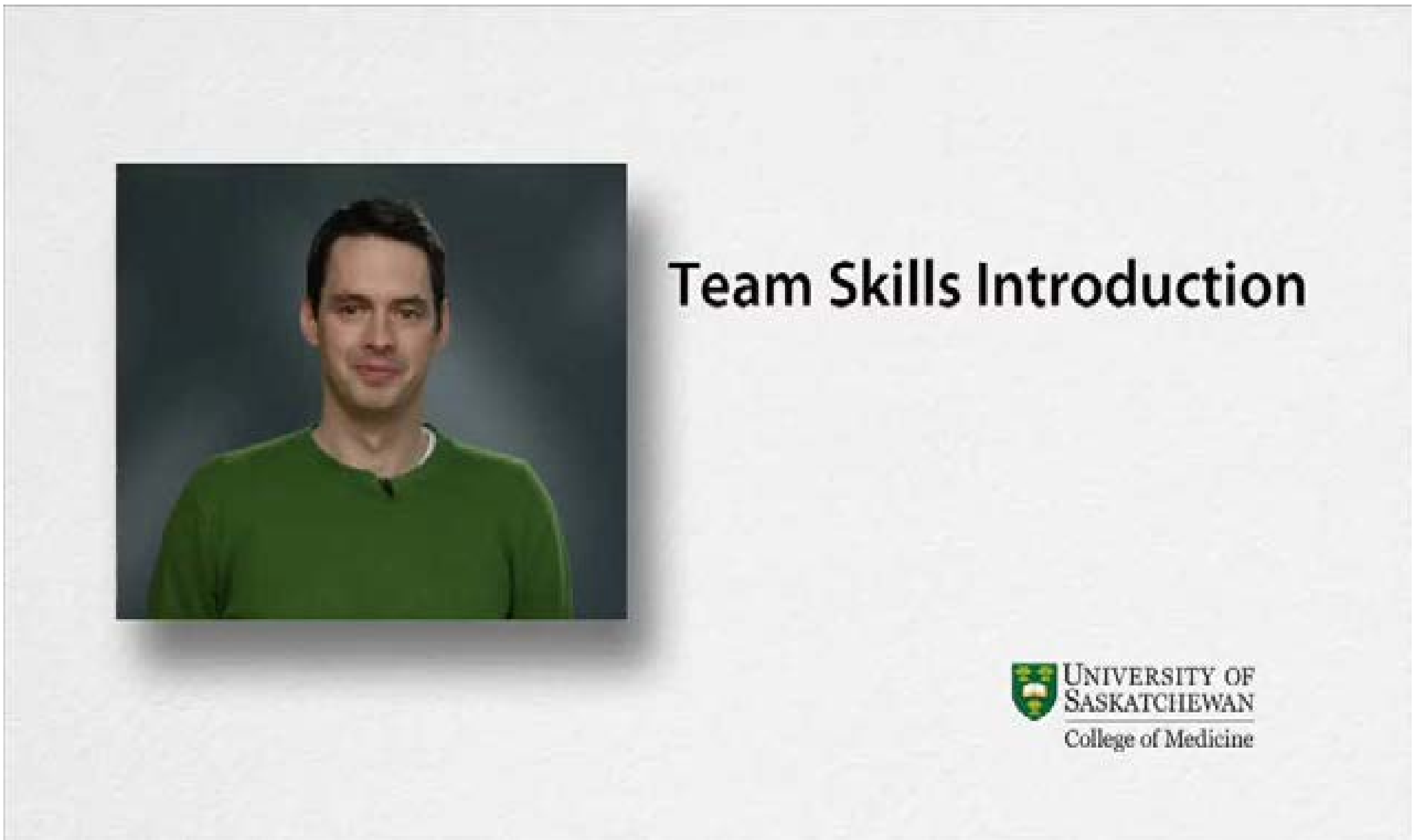

Situational Awareness

Situational awareness is simply being aware of what is going on around you and anticipating what needs to be done. (Endsley 1995). Wrights describes it as seeking out information and recognizing and understanding the significance of this information for patient care needs and team goals. Having team awareness of what other professionals contribute to patient care in the setting of a rapidly changing hospital environment is necessary for the team to develop shared mental models, for patient centred outcomes and the processes to achieve these outcomes. Being aware and exchanging information will bridge silos, prevent errors and ensure patient care needs are met.

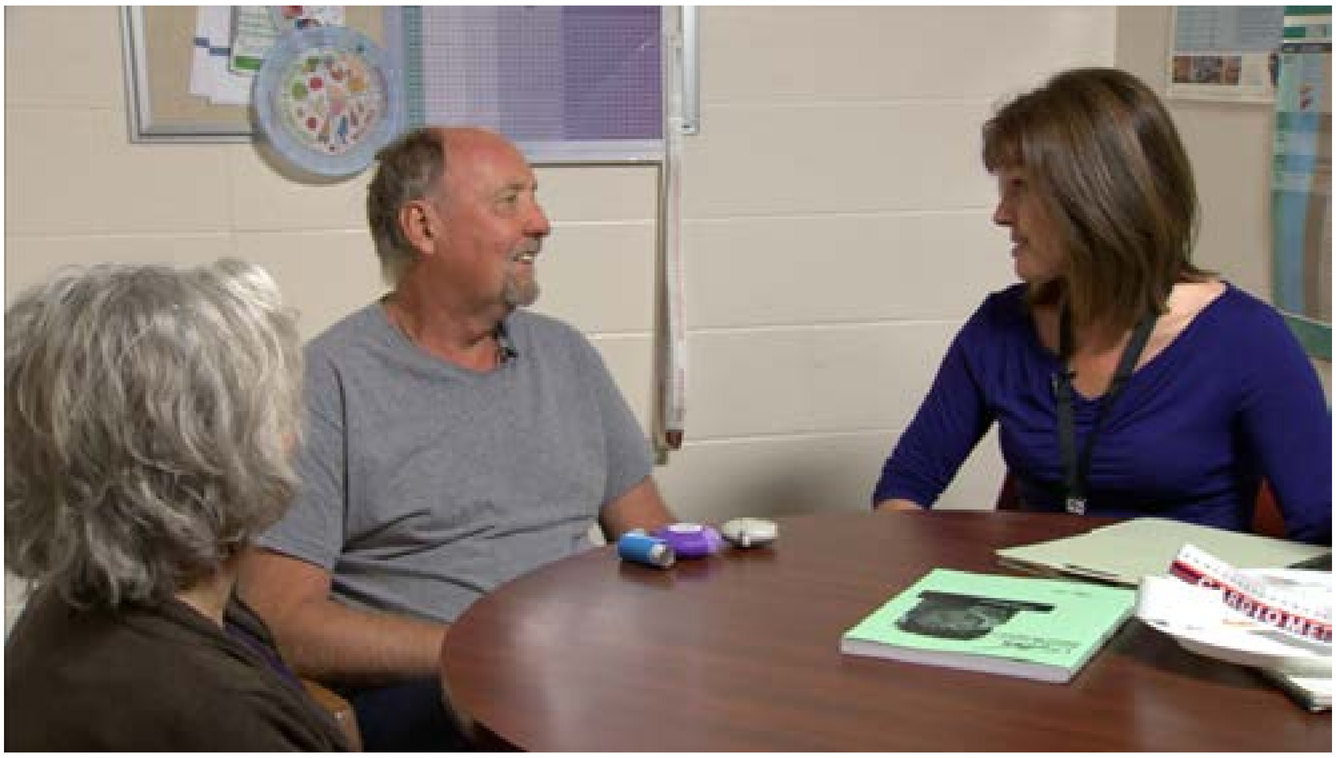

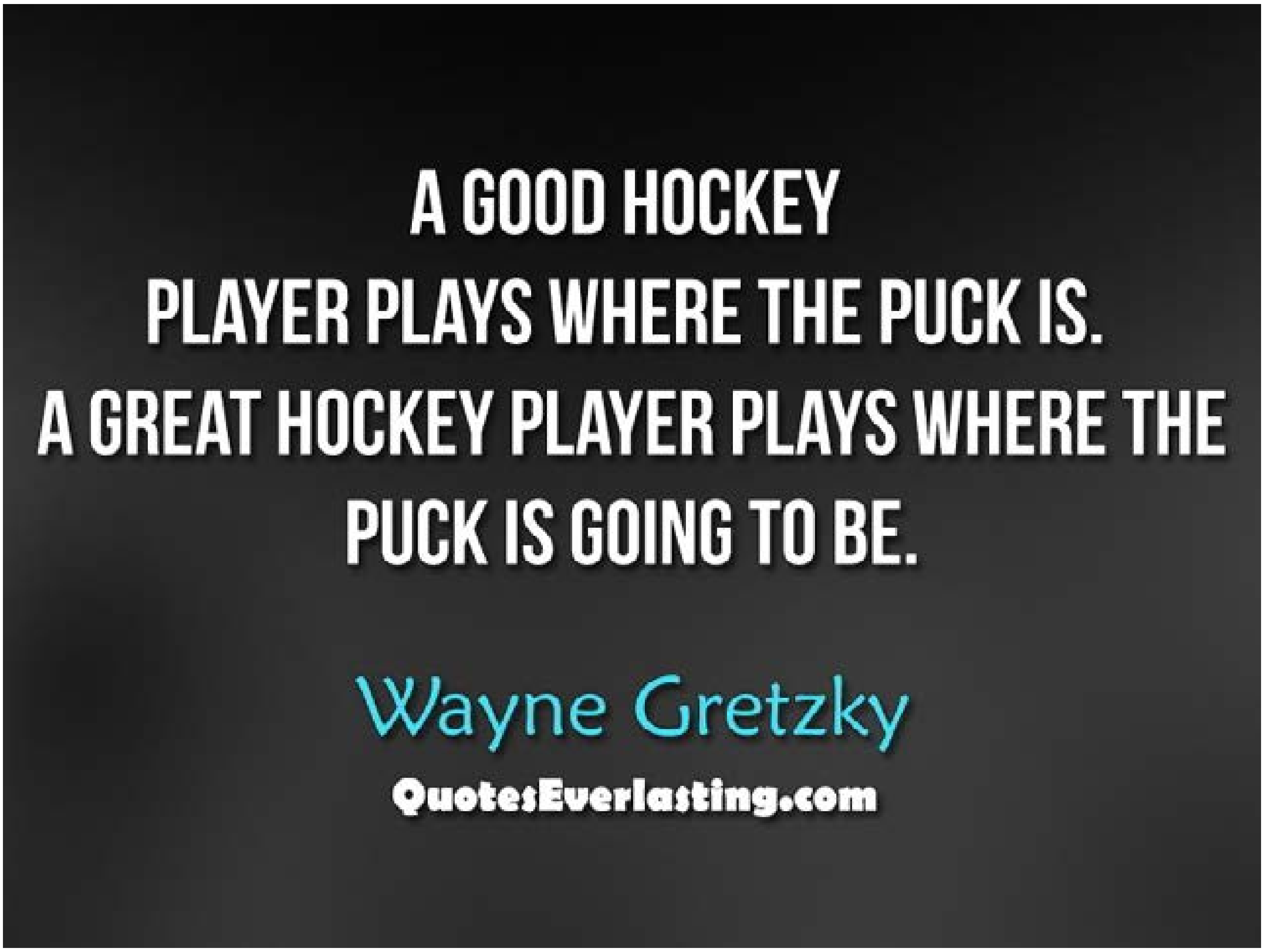

Photo "[A Good Hockey Player Plays Where the Puck Is](https://flic.kr/p/ehVvk9)*" by Quotes Everlasting is licensed under* [CC 2.0](https://flic.kr/p/ehVvk9)
https://flic.kr/p/ehVvk9

Shared Mental Models

Shared mental models are the common goals for patient care. Westi (2010) defines shared mental models as joint understanding of the tasks and roles necessary to facilitate coordination of care and meet patient's identified goals. Shared mental models provide an opportunity to break down the silos that occur between disease-based care and different health professions. Not every individual health care profession will have the same mental model or cognitive map of individual patient care needs. Each individual healthcare professional's mental model is incorporated into a shared or team mental model of care. This provides the teams with strategies to know who can and needs to contribute each role for the team to be successful at accomplishing the patient's goals of care. Smith Jentash (2005) states that having shared expectations of outcomes of care (both task and team roles) allows for more comprehensive care with less overlap and fewer gaps.

Situational Awareness and Shared Mental Models are skills that are dependent on one another to achieve understanding of the task; awareness of the expertise and roles available to meet patient care needs for chronic disease and identified social needs. Neither Situational Awareness nor Shared Mental Models can be applied to effective interprofessional care without effective communication (Westi 2010).

SBAR (Situation, Background, Assessment, Recommendation)

SBAR is a standardized communication tool that neutralizes differences in knowledge and hierarchies, and addresses uncertainty of roles and responsibilities. When describing a clinical situation or question to another healthcare professional, ordering or grouping the information by the SBAR headings (situation, background, assessment and recommendation) standardizes communication and provides a better alternative to the often-used 'hint and hope' model. SBAR provides a structured or standardized approach to communication among healthcare professionals that links situational awareness and shared mental models resulting in effective teamwork. Communication is more effective if there is a common style or structure everyone is familiar with and a structure that neutralizes hierarchies (Westi 2010).

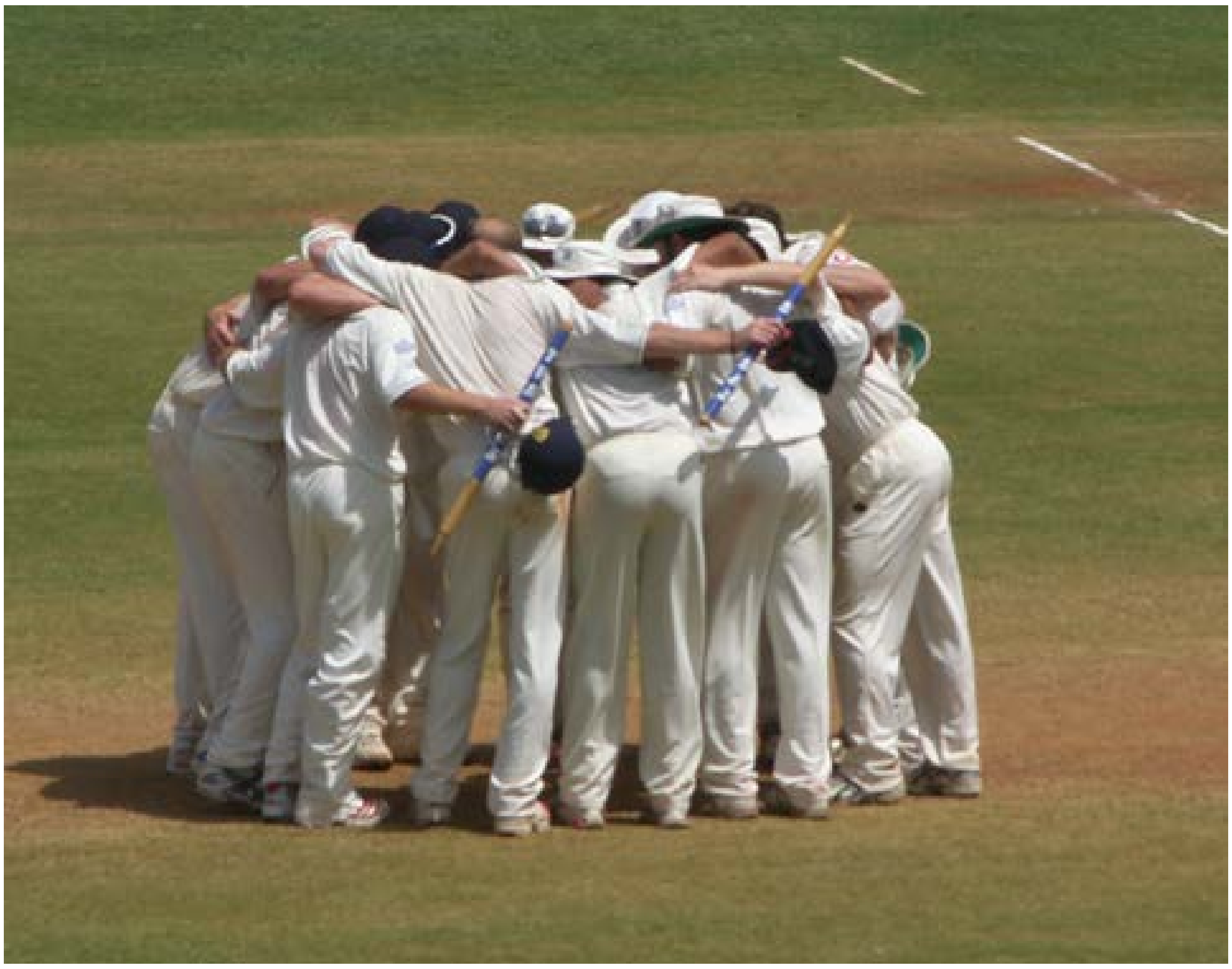

Image "Victory Huddle" is in the public domain:
https://commons.wikimedia.org/wiki/File:Victory_huddle.jpg

When you begin to use **SBAR**, you will find that by describing the situation, you are encouraged to express your clinical concern or question and to describe your role on the team for this clinical item. For example, you as the pharmacist could say that Mr. Sim is not certain how to use his inhaled medications correctly.

Background is often a challenging step to select information that will concisely provide background or context. This will come with practice. For this situation, the background could include that there are new inhaled medications for COPD that Mr. Sim has not previously used.

Your assessment provides the bridge between individual professional silos. The assessment also provides the rationale for your subsequent recommendation. **SBAR** provides a mechanism for speaking up and expressing your concerns that in this framework can be more readily understood and acknowledged by the team.

The recommendation is often the most difficult part as you put your ideas forward to the rest of the team; however, the information you provided through the other components of **SBAR** provide the context and rationale for your contribution to team function. As everyone uses the same structure for communication, a level playing field and effective communication is provided. For further examples of **SBAR**, please see the reference section.

In Summary: Team skills are necessary for Healthcare of chronic multisystem diseases

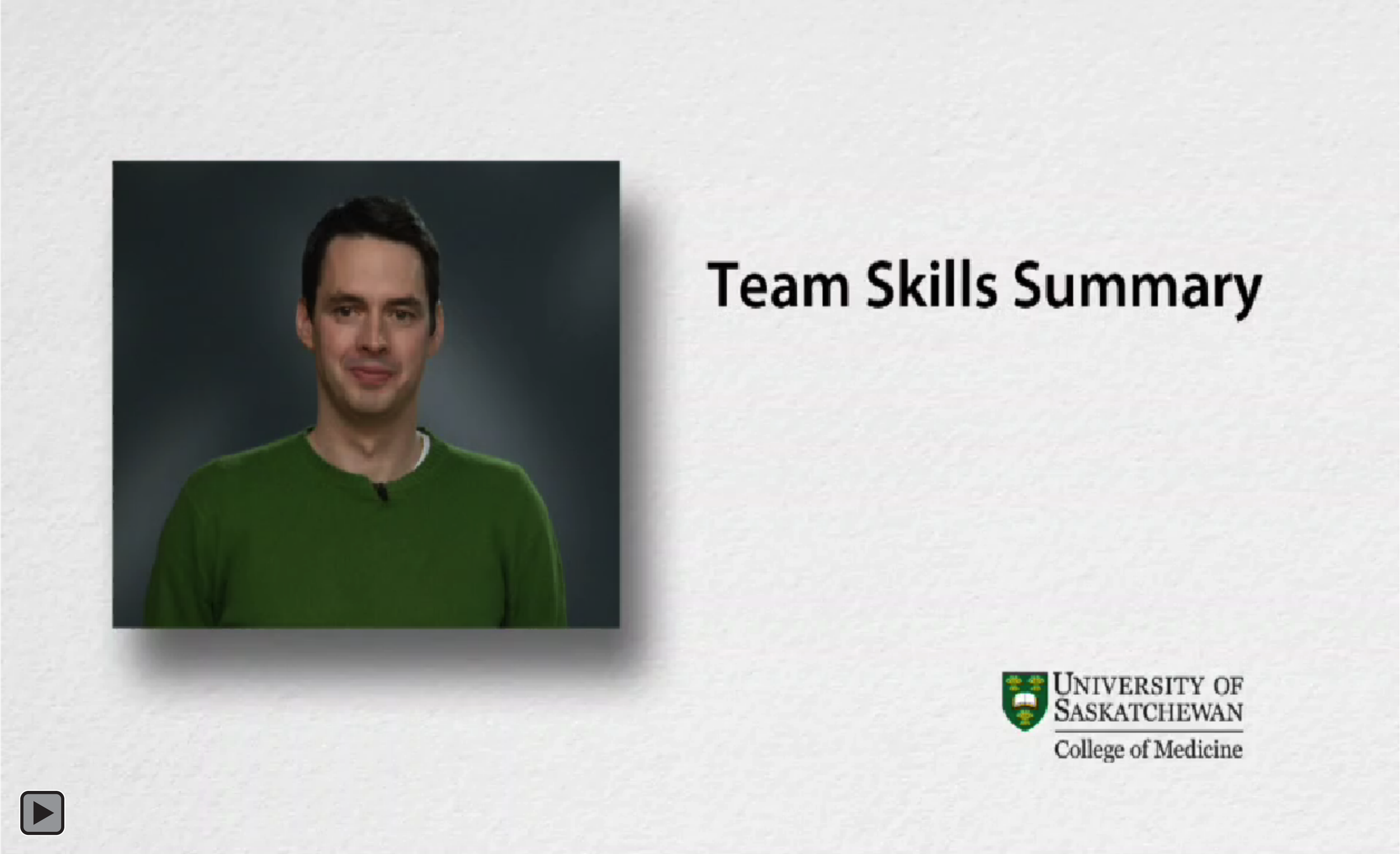

The overall goal of team care is to contribute expertise, share the workload of patient care and identify and meet patient care goals for each individual patient.

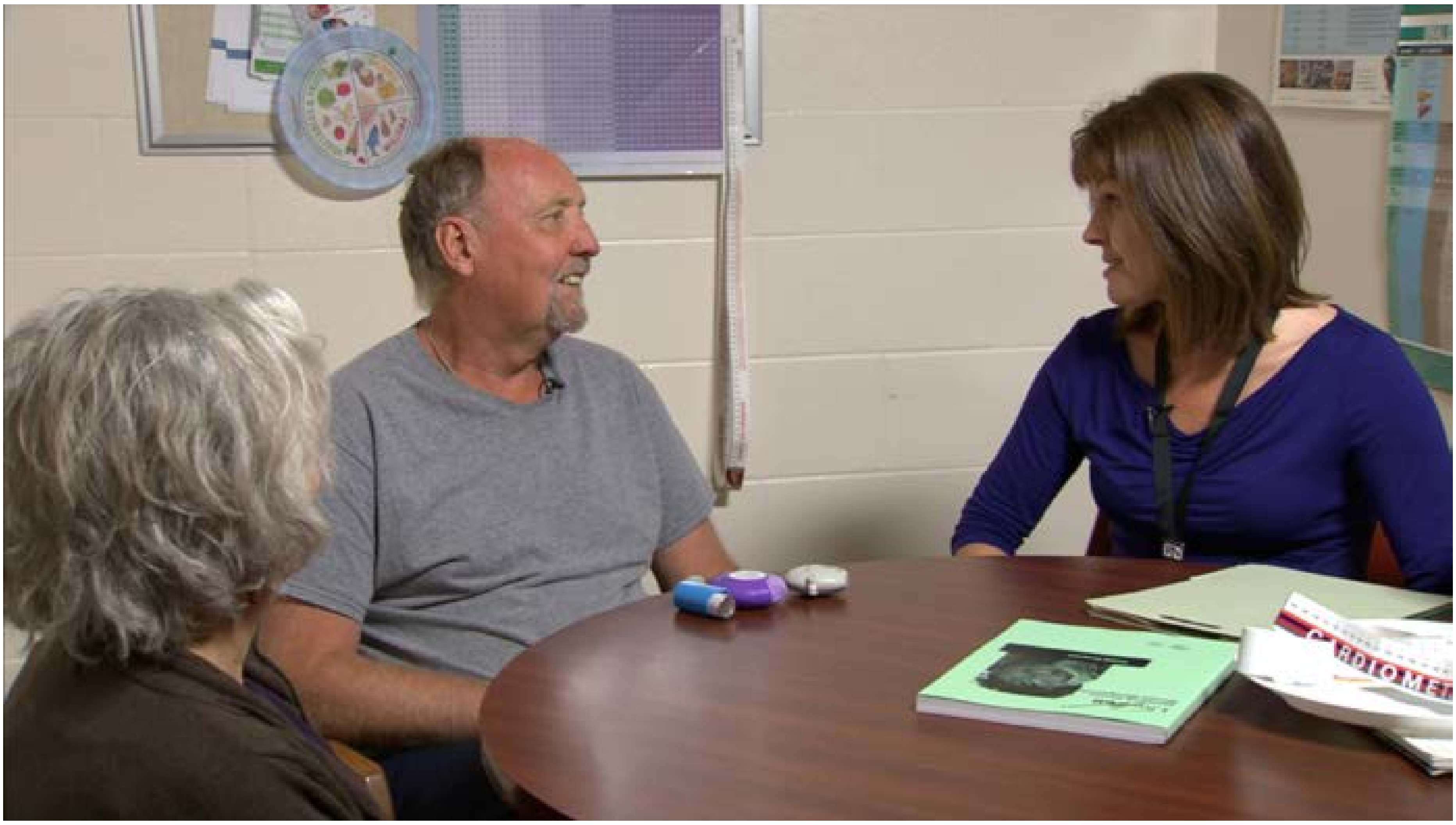

Chapter 3: Clinical information; a not so good discharge

As we reflect on the importance of inter professional care, team skills, and multi system chronic disease management, let's meet Mr. Sim and review his clinical information in more detail. Mr Sim is currently in hospital and is preparing for discharge home.

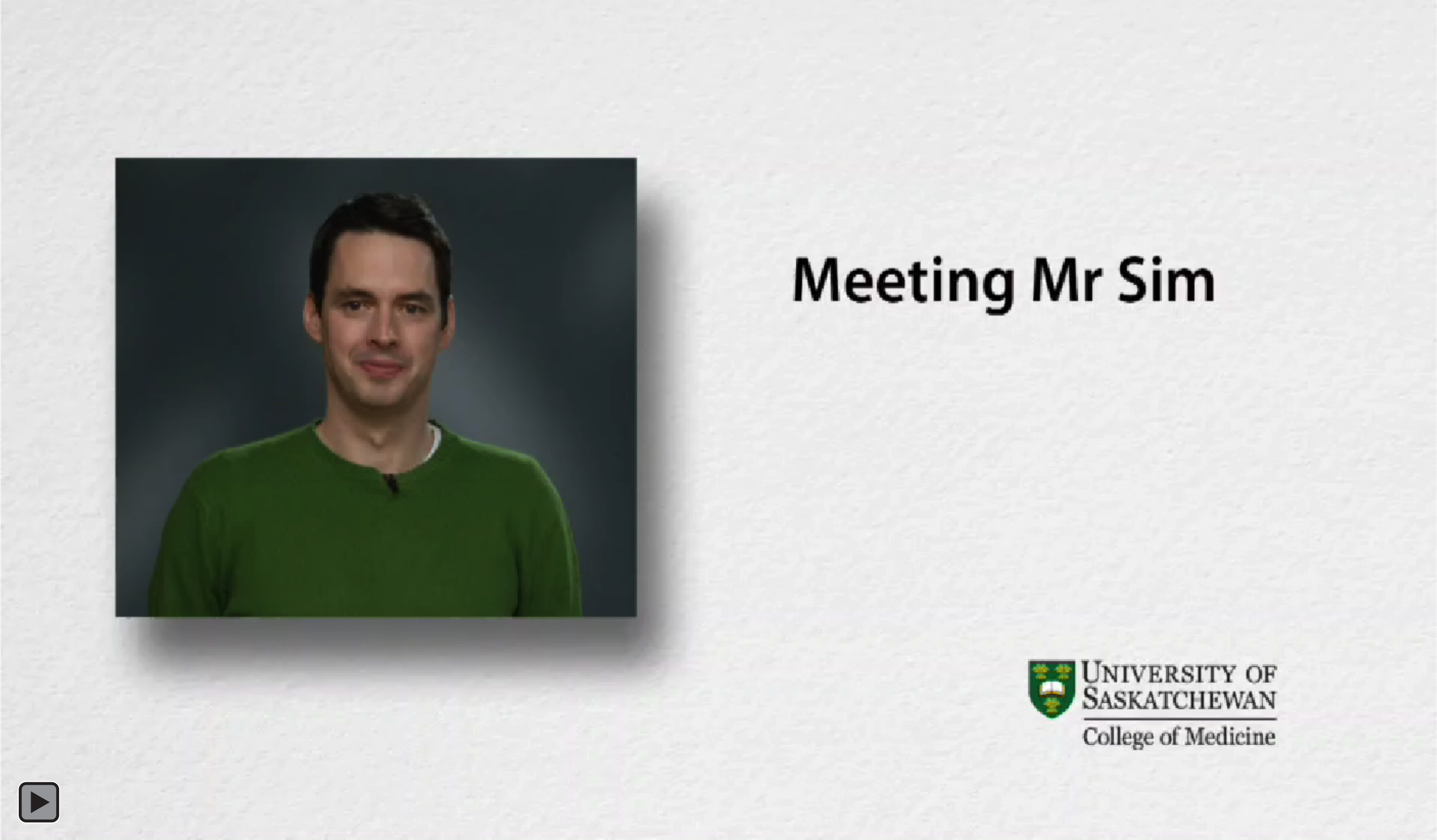

The case: Mr Sim's hospital Chart

Mr. Sim is a 64 year old gentleman with moderate COPD who has been in hospital for 5 days with his first ever COPD exacerbation requiring hospitalization. There is a clinical chart available for your review.

Just as you would in hospital when meeting a patient for the first time, read through the clinical information for Mr. Sim - his admitting data base, medications, medical flow sheet and progress notes of various healthcare providers. This information is available in appendix 1. When you are done reviewing the chart there will be a link that will connect you back to this chapter.

Task 1: Identifying Mr. Sim's care goals and the healthcare professional team

Now that you have reviewed his chart, let's meet Mr. Sim as he discusses his concerns regarding discharge with Vicki, a Nurse from the community-based COPD program. As you watch this video, make your own list of discharge items that need to be addressed before hospital discharge. Identify the healthcare professionals you think will need to be consulted to address these issues. In the group section (Chapter 7) of the ebook you will be asked to discuss your list with your colleagues.

An example of how to record information is provided. Keep your list handy, as you will need to refer to it in chapter 7 as you practice applying SBAR communication to Mr. Sim's discharge planning (group task 4).

**Example List of Discharge Items**	
Discharge Concern	***Healthcare Professional(s)***
Medications: When and how to use COPD inhalers	

Now lets watch the video of Mr. Sim talking with a healthcare provider:

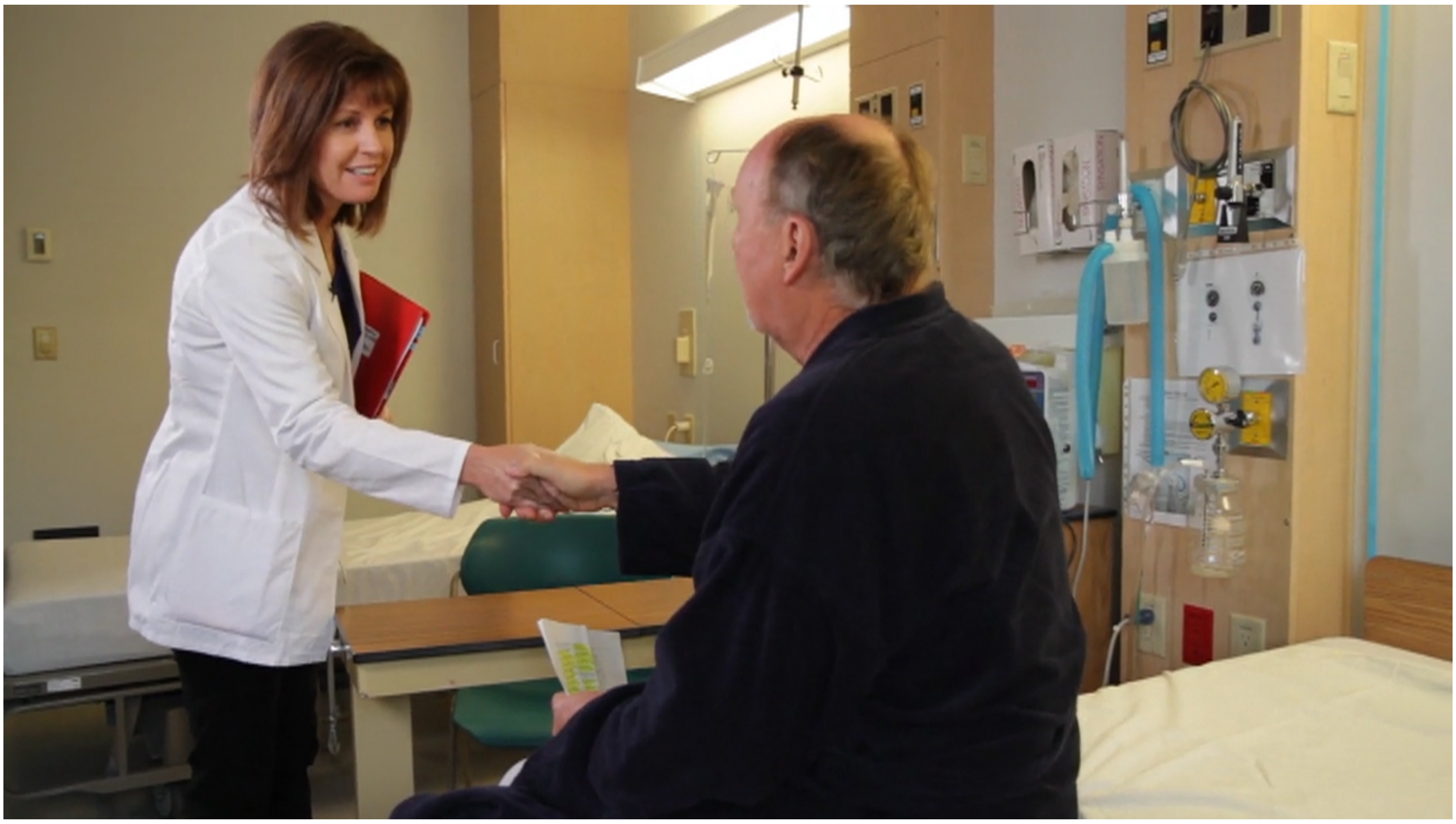

- Based on the paper chart and this video, list what you identify as medical and personal discharge issues for Mr. Sim. Prepare your list similarly to the example in appendix 2. Include the clinical or social problem and the healthcare professionals who need to be involved to addresses each of the issues.
- For an example of a completed care goal list please see appendix 2. The list of care issues and health care professionals identified is not all-inclusive and you may have identified different items. There will be a link that will connect you back to this chapter when you are done reviewing the table.

Task 2: Reflecting on hospital discharge from your own professional experience

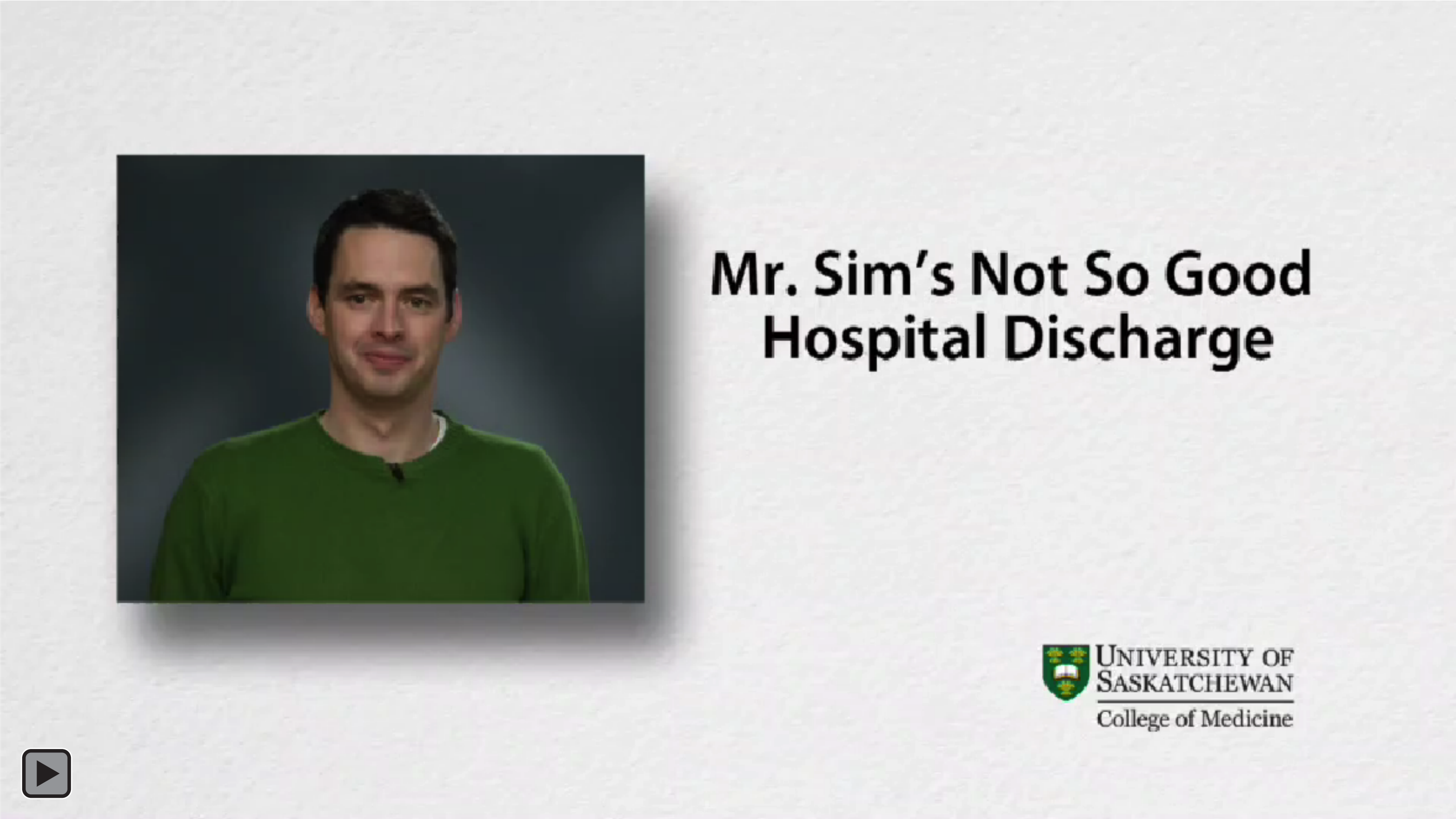

In the next video we see that Mr. Sim has been released from the hospital. He shares with his wife, his perception of the discharge.

- After watching the video, reflect for a moment on hospital discharges you have participated in. How challenging is it to have a good discharge in which the transition home goes according to everyone's expectations?
- Now reflect on individuals you have seen with problems arising from the discharge, sometimes resulting in patient harm or hospital re-admission. What do you think are important skills and attitudes for healthcare professionals working as a team participating in a hospital discharge?

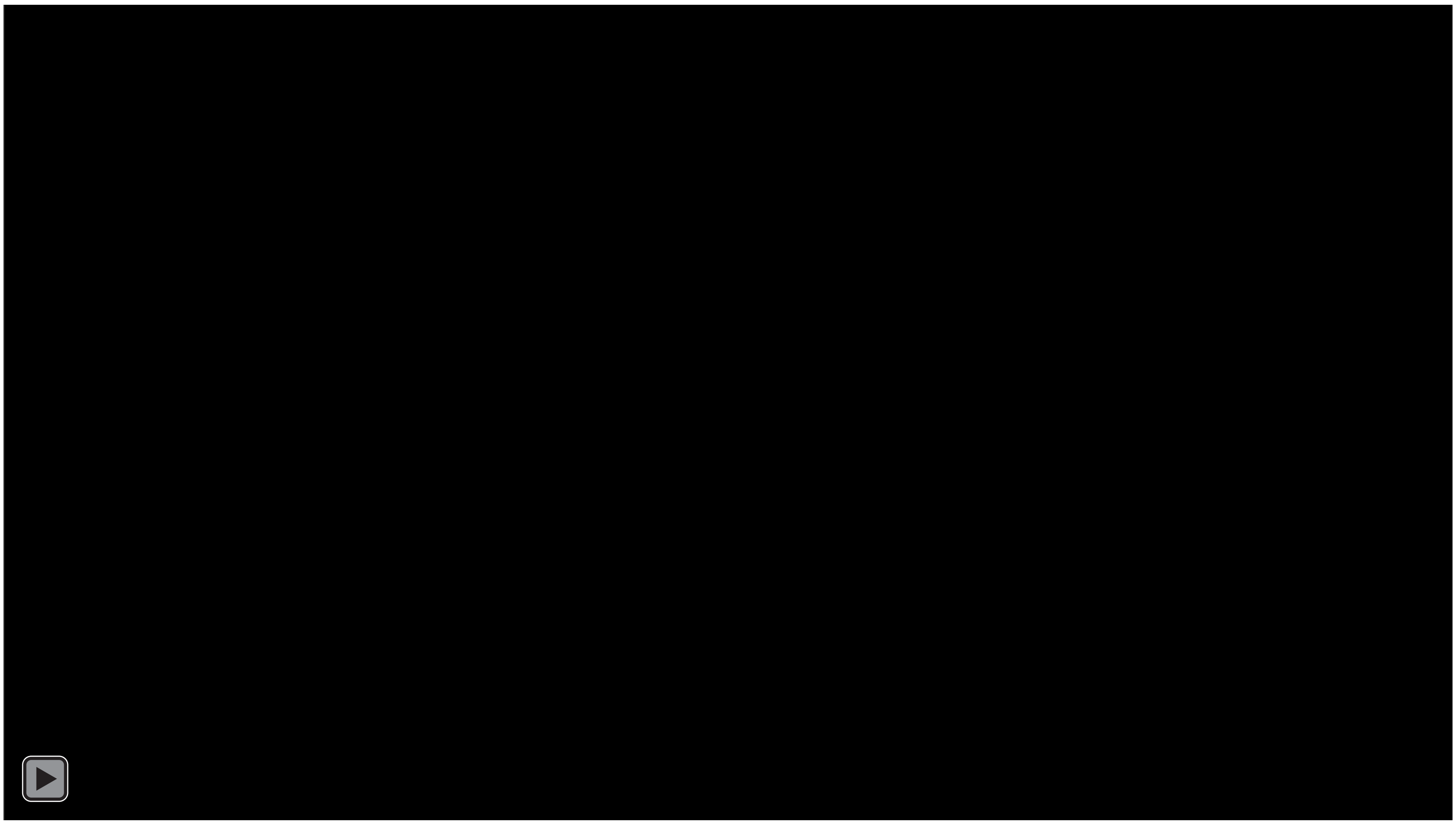

Chapter 4: Interprofessional discharge planning: applied use of Situational Awareness, Shared Mental Models, and SBAR

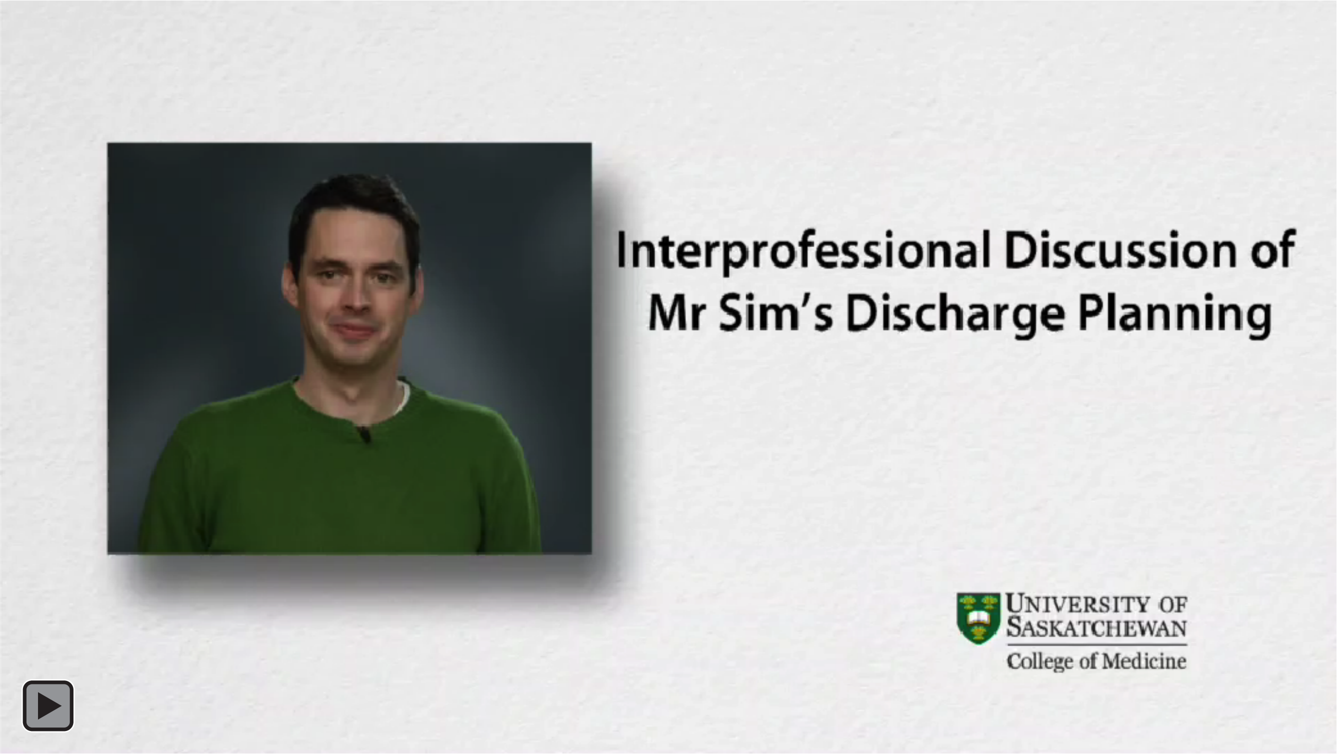

Task 3: Identifying Examples of Situational Awareness, Shared Mental Models and SBAR

In the following video of Mr Sim's discharge planning, the team will only be reviewing some of the discharge issues pertaining to Mr. Sim. You may have identified other relevant issues not discussed in this brief video clip that demonstrates interprofessional team skills. You can review your own list of Mr. Sim's care goals or review appendix 2 for a list of care goals.

While watching this video please reflect on the following:

- Were situational awareness, shared mental models and SBAR demonstrated as team skills in this video? What were some examples you identified?
- How would these techniques apply in your own practice setting? Could they be used to bridge disease based and professional silos? Will they facilitate inclusion of patient's choices in planning healthcare and address the clinical as well as the social needs of patients?
- Would patient care needs and expected roles to meet these needs be better communicated among healthcare team members using SBAR?

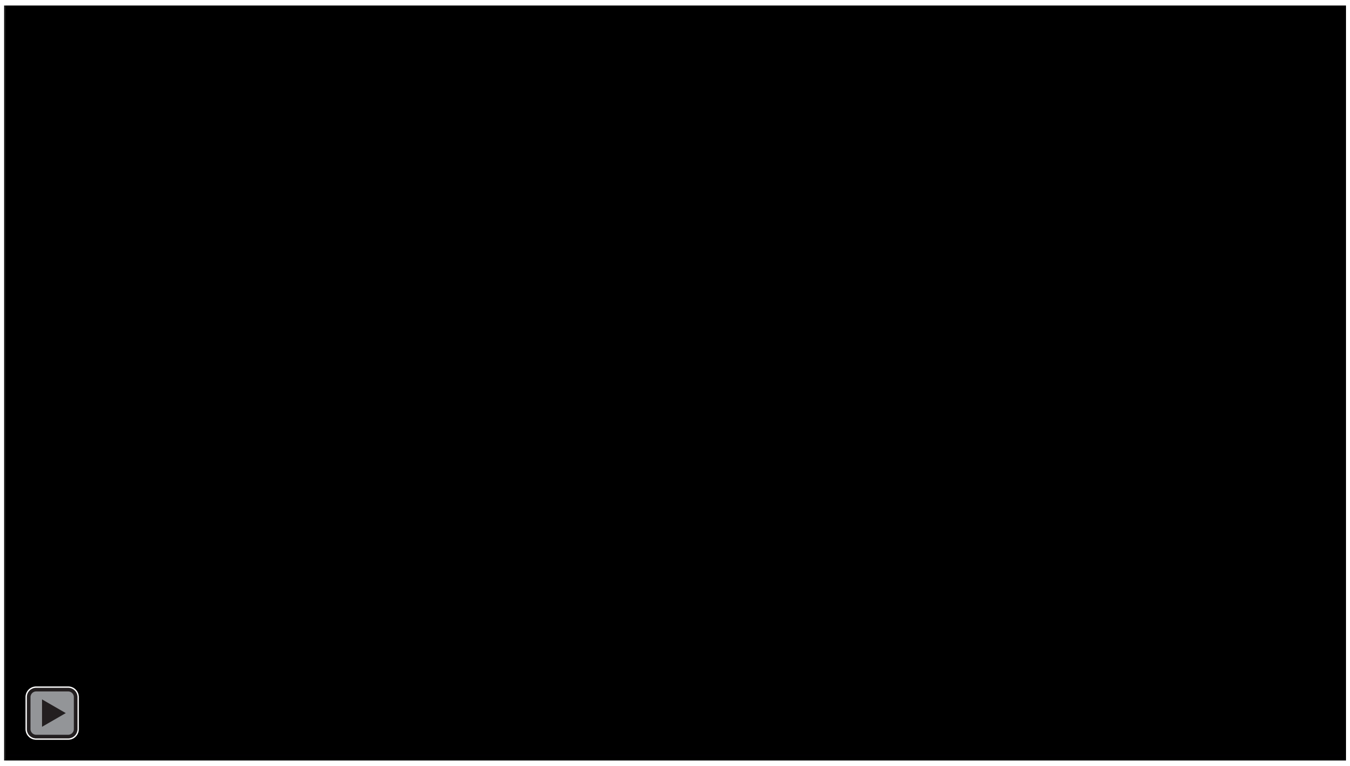

Chapter 5: Mr. Sim in the community - the outcome of team skills

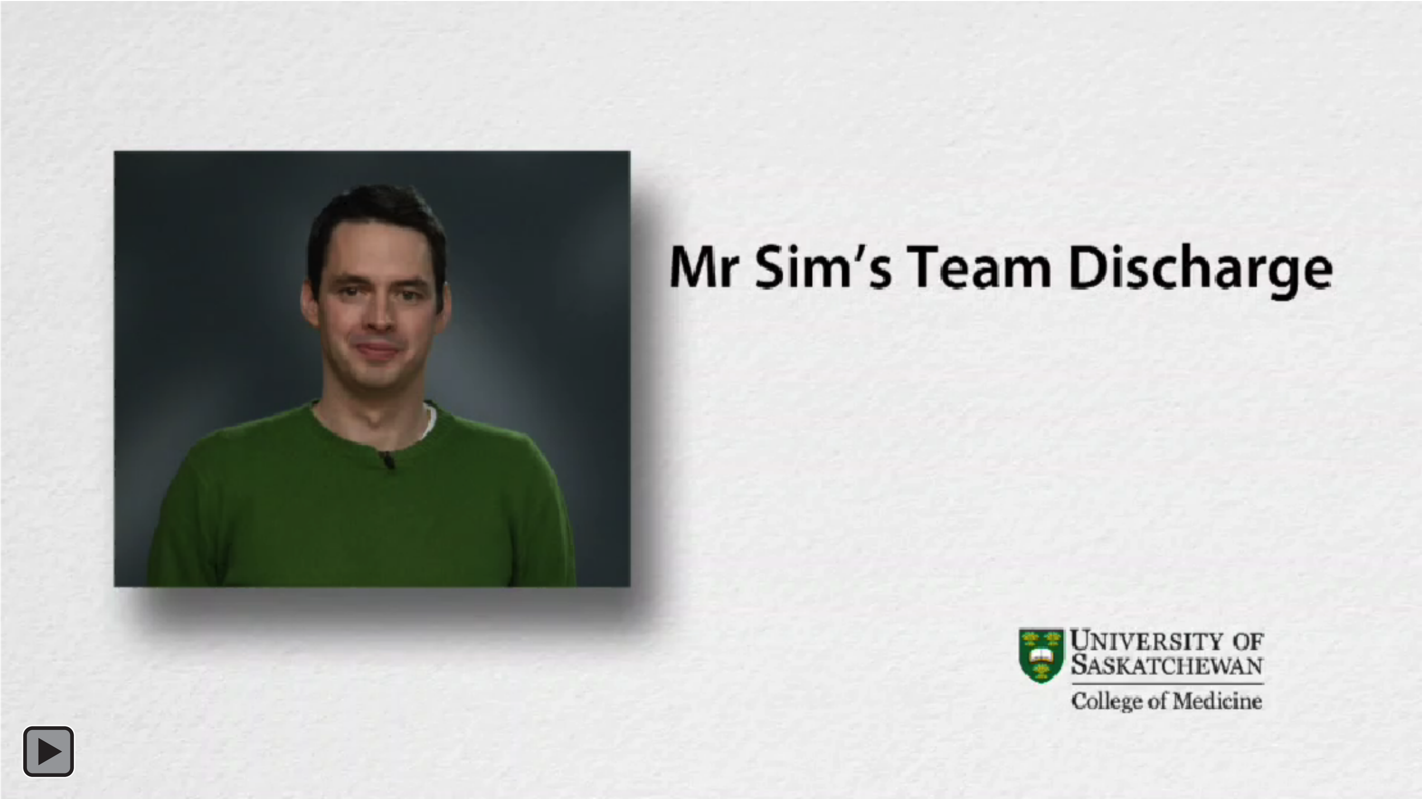

Task 4: Applying SA SMM and SBAR to discharge Planning

In the next video we see that Mr. Sim has been released from the hospital. He shares with his wife, his perception of the discharge.

- While watching this video decide if you think situational awareness, shared mental models and SBAR would result in more comprehensive discharge planning with both medical and social needs addressed.
- How would this happen?
- Would patient care needs and expected roles to meet these needs be better communicated among healthcare team members using SBAR? How would SBAR facilitate this?

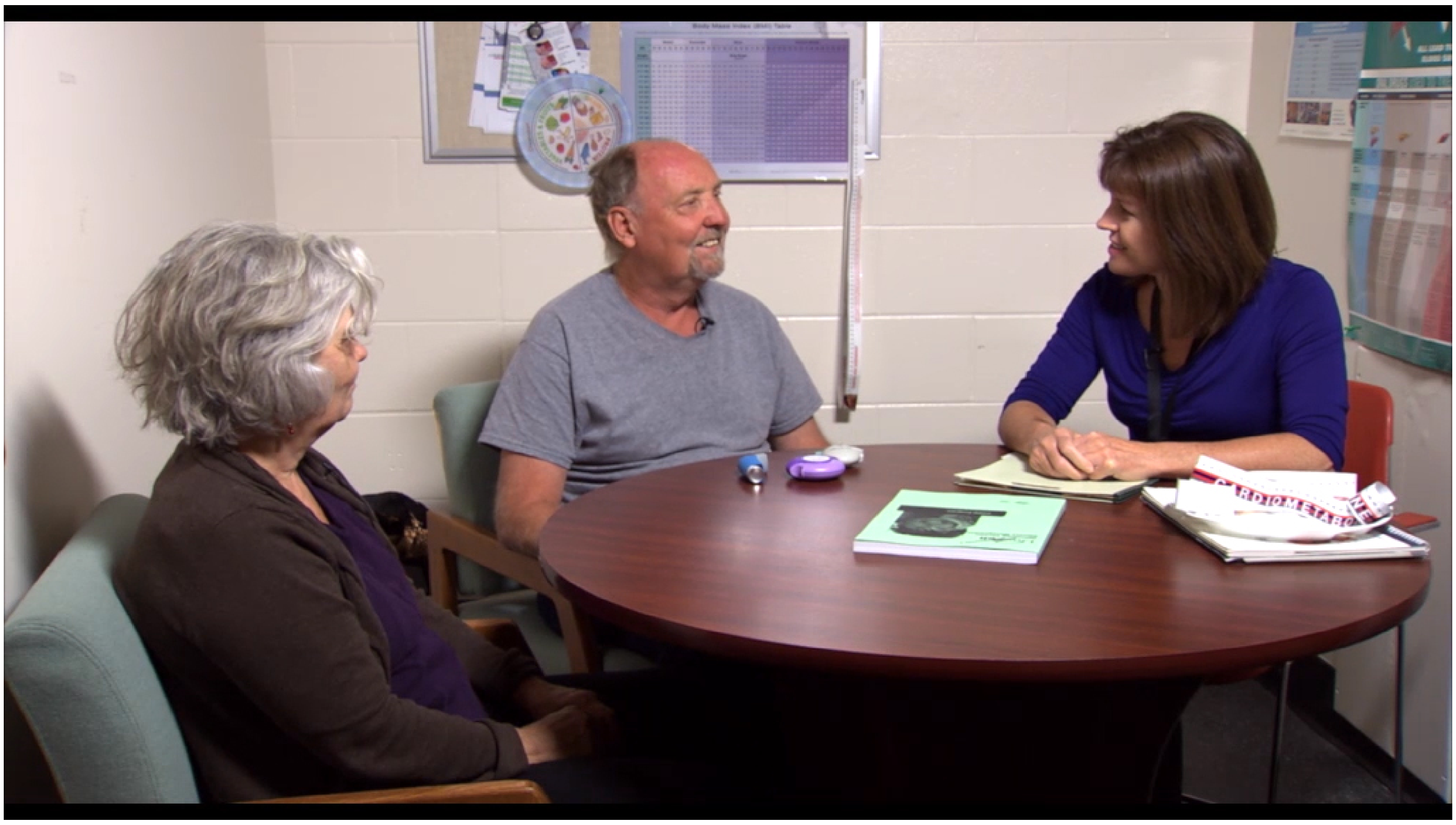

Now that you have become familiar with Situational Awareness, Shared Mental Models and SBAR, will you begin to use these team skills in your clinical environment?

Chapter 6: Conclusion

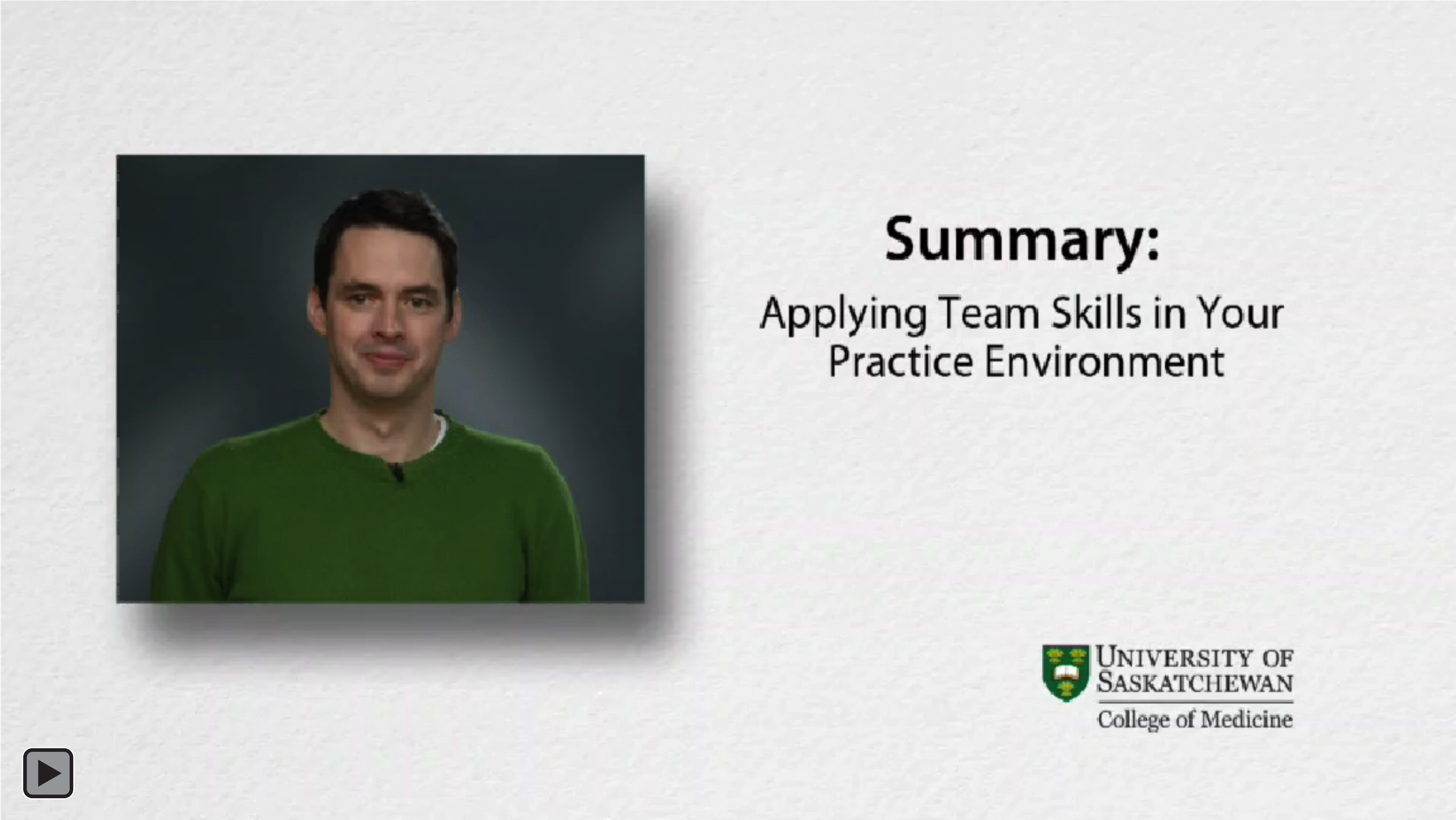

SBAR, Situational Awareness and Shared Mental Models have been described and demonstrated as team skills that can facilitate interprofessional care. Through your reflections and observation of these concepts, identify what you found valuable from this session and what you will take to apply in your learning or practice environment.

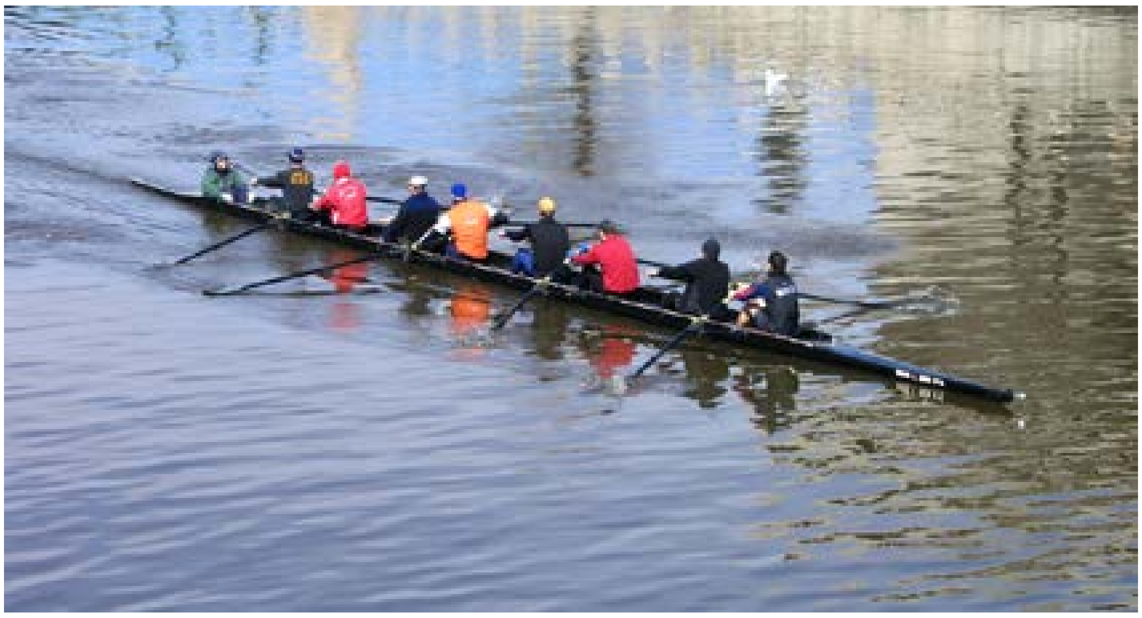

Image "Rowers" by Michael Pereckas licensed under CC 2.0

Task 5: Taking these team skills back to your Practice or Learning environment

The next step when taking these concepts back to your working or learning environment, is to share them with your colleagues.

- Identify some ways that you could introduce SBAR as a communication tool into your environment. How will you tell others about situational awareness and shared mental models?
- If you had a few moments during a coffee or lunch break, how would you summarize what you have learned and explain it to your coworkers.

Thank you for participating in this introduction to interprofessional team skills in an acute care setting. We each bring our own unique knowledge, skills and perspectives to patient care and all are valuable. The challenge is to bring the individual expertise together and bridge professional and disease based silos to provide integrated care that includes the patient as a team member. As we have reviewed, there are challenges to integrating expertise and experience when providing patient care, but these team skills have been developed to facilitate the processes and create an environment of interprofessional practice which includes patient and families as team members.

At the end of the ebook, following the appendices, you will find the references and additional links to Situational Awareness, Shared Mental Models and SBAR as an approach to interprofessional practice to meet healthcare needs.

In chapter 7, the learning tasks you completed as an individual learner are provided in the context of a group discussion. Group discussion with members of your own profession and especially members of other healthcare professionals provide opportunity for different perspective and an opportunity to apply the team skills in a group setting.

Chapter 7: Group Learning and Discussion

This section provides opportunity for you to discuss within a small group your reflections and learning from reviewing the interprofessional ebook content individually. Please return to the ebook at anytime to review relevant information. Links are provided to the tasks and associated videos with clinical information for Mr. Sim and the interprofessional team. Your e-reader should provide you with chapter and subheadings that you can use to navigate through the ebook content.

Group Task 1: Identifying Mr. Sim's care goals and the healthcare professional team.

Using the list of care goals and the related health care professionals you identified when you reviewed the ebook (Task 1 Chapter 3), discuss as a group what you identified as his main care goals. If new care goals were introduced by other members of your discussion group, please review why this is an identified care goal and which healthcare professional would most commonly address this need. If you need, review the video of Mr. Sim discussing his clinical condition with the healthcare professional.

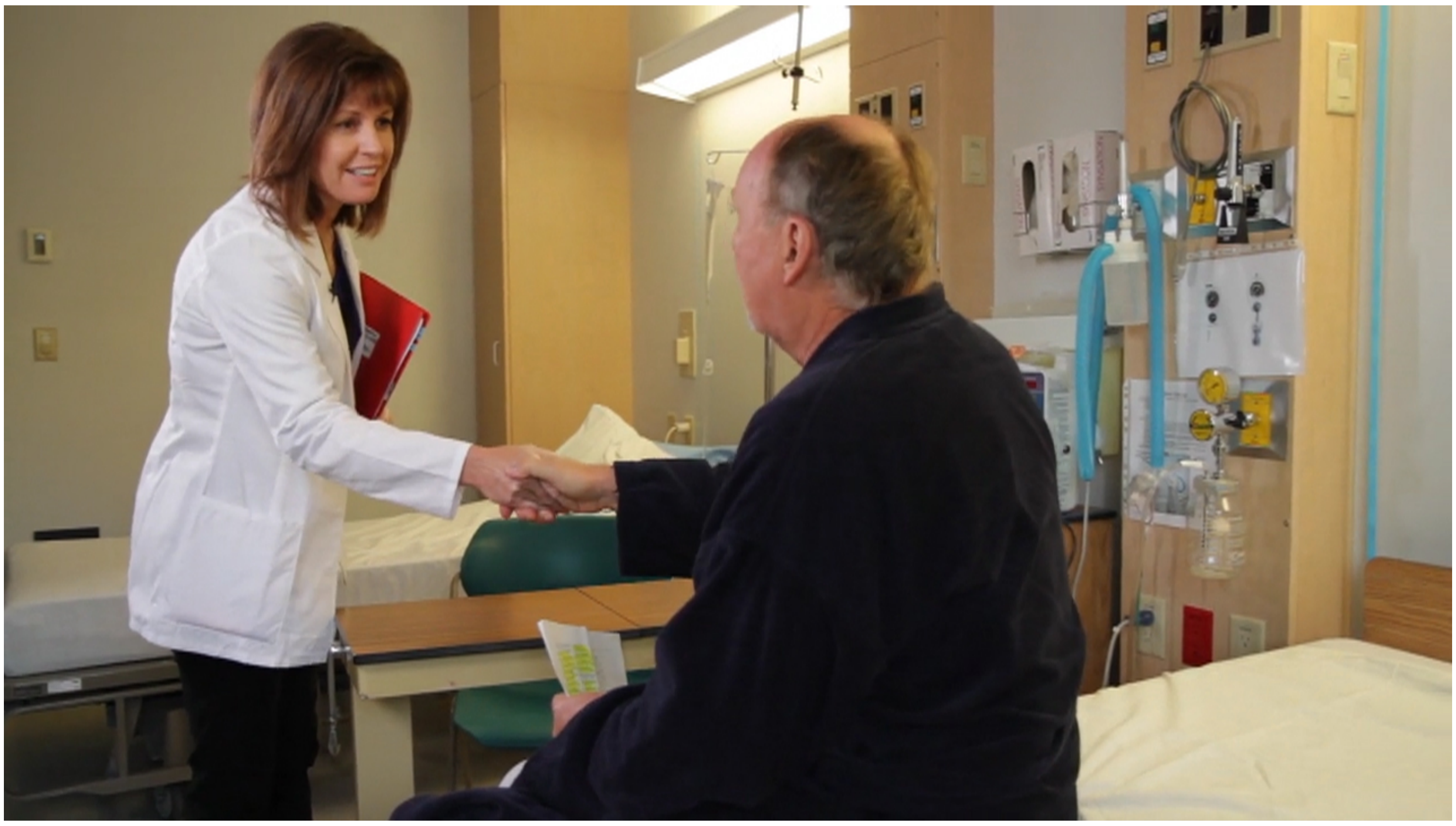

Group Task 2: Discussing positive and negative discharge experiences.

As a group, reflect on Mr. Sim's not so good discharge and recall your thoughts on what you have experienced that has gone well and not so well with hospital discharges, or transitions to another care environment or home. Discuss your thoughts and ideas as a group.

Group Task 3: Identifying examples of situational awareness, shared mental models and SBAR.

As a group, replay the video of the **interprofessional healthcare conference**. As you watch the video, review your list of examples of team skills. Discuss as a group examples you identified of situational awareness, shared mental models and SBAR and how you think each of these contributed to the team's work as they discussed Mr. Sim's discharge.

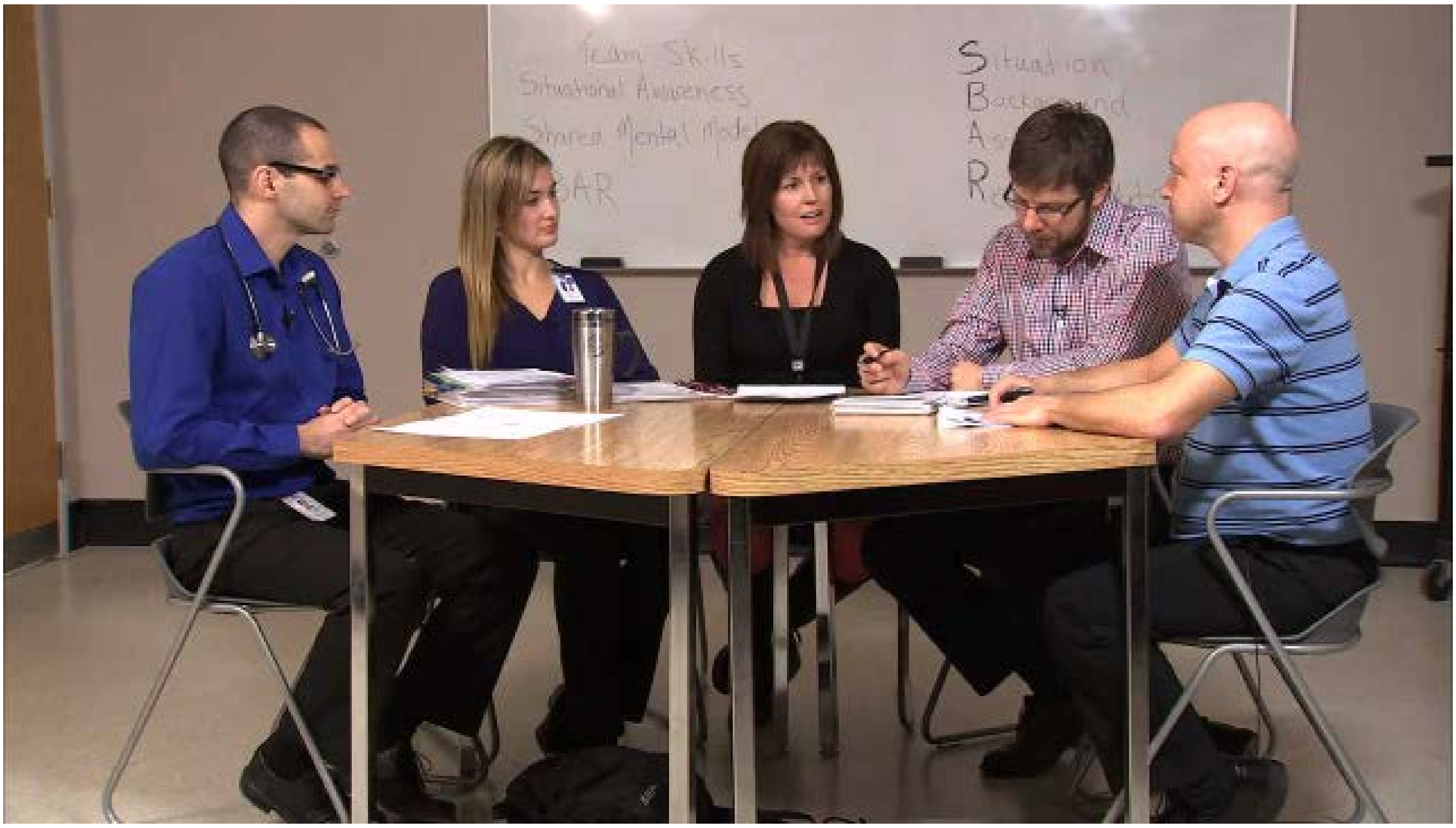

Group Task 4: Practicing SBAR

Let' take a few minutes to practice using the communication technique SBAR. Look at the items you identified regarding Mr. Sim's discharge. Each member of the group should identify one item and using SBAR present and discuss the issue with the rest of the group. As you work through using SBAR, discuss as a group what you think would be incorporated into each component of the technique. What are some challenges to each component? How do you feel about committing to a recommendation to the team using SBAR? Discussing each component of SBAR will help you learn this new communication skill. When you have finished this exercise please move on to the next chapter. Appendix 2

Group Task 5: Taking these team skills back to your practice or learning environment

As a group, discuss how you would summarize what you have learned regarding team skills and how you would describe the team skills reviewed in the ebook to your coworkers. Pair up with someone in the group and briefly explain one of the team skills presented in the ebook to your partner and describe how you think it should be applied in practice.

Thank you for participating in this introduction to interprofessional team skills in an acute care setting. We each bring our own unique knowledge, skills, and perspectives to patient care and all are valuable. The challenge is to bring the individual expertise together and bridge professional and disease based silos to provide integrated care that includes the patient as a team member. As we have reviewed, there are challenges to integrating expertise and experience, but these team skills have been developed to facilitate the processes and create an environment of interprofessional practice which includes patient and families as team members.

Following the appendices you will find the references and additional links to situational awareness, shared mental models and SBAR as an approach to interprofessional practice to meet healthcare needs.

Appendix 1: Mr Sim's Chart

Mr. Sim

SASKATOON HEALTH REGION
Saskatoon, Saskatchewan

☐ RUH ☐ SCH ☐ SPH Other ________

MEDICAL FLOW SHEET - DAYS

Page 1 of 2
Date: Day 2

CNS: *Normal Assessment* ➤ *Alert, Up ad lib, Calm, Oriented to Person, Place, Time, Follows Commands, Speech Clear, No Pain, Sensation Intact*

☑ **WNL** (within normal limits) ☐ **WNL except:**
LOC: ☐ restless ☐ drowsy ☐ lethargic
☐ aphasic ☐ dysphasic ☐ unresponsive
Disorientation:
☐ person ☐ place ☐ time
☐ confused ☐ inappropriate
Pain ________
☐ see MAR
☐ Epidural ☐ PCA ☐ HDC
Mobility: ☐ with assist ☐ bedrest
Activity/Tolerance ________
Movement and Sensation ________
Other ________

CVS: *Normal Assessment* ➤ Skin: Color Within Normal Limits, Warm & Dry, Heart Rhythm Regular, Peripheral Pulses Present, No Edema

☑ **WNL** ☐ **WNL except:**
Color: ☐ pale ☐ dusky ☐ cyanotic
☐ flushed
Temp: ☐ cool ☐ hot ☐ diaphoretic
Peripheral Pulses ________
Edema mild edema
Leg/Ankle Exercises q ________
IV/HDC Site Condition good
Other ________

PSYCH/SOC: *Normal Assessment* ➤ *Behavior Appropriate, Stable Emotional State, Coping Patterns Appropriate, Family Involved*

☐ **WNL** ☐ **WNL except:**
Family/Visitors wife in to visit
Coping Pattern worried about going home.
☐ See Teaching Plan ☐ See Discharge Plan
Other ________

RESP: *Normal Assessment* ➤ *Respirations Unlaboured & Symmetric, Regular Rhythm & Depth, Cough Not Productive, Not Intubated*

☐ **WNL** ☐ **WNL except:**
Respirations: ☑ SOB ☐ SOBOE
Abnormal Breath Sounds wheezes bilaterally
Cough productive
DB&C q ________
Secretions green/yellow
Suctioned ——
ETT/Trach Care/Site ——
Other increased effort. SOB moving in bed.

GI/GU: *Normal Assessment* ➤ *Abdomen Soft, No Nausea or Vomiting, Tolerating Oral Diet, No Diarrhea or Constipation, Voiding Without Difficulty, Urine Clear, No Bladder Distention, No Vaginal/Penile Drainage, Continent of Urine/Stool*

☐ **WNL** ☑ **WNL except:**
☐ Nausea ☐ Vomiting ☐ NPO
NG Suction ________
Enteral Feeding ________
PGJ Site/Drsg ________
Abdomen: ☐ firm ☑ distended ☐ tender
☐ abn. bowel sounds ________
Stool: ☐ diarrhea ☐ constipation
Incontinent of: ☐ stool ☐ urine
Catheter ________
Vag/Penile Drainage ________
Other SOB when eating

INTEG: *Normal Assessment* ➤ *Skin, Clear, (free of Rashes, Reddened Areas, Open Areas or Bruises*

☑ **WNL** ☐ **WNL except:**
Skin Care/Condition/Positioning q ________
Incisions/Dressings/Wounds/Drains ________
Other ________

Assessment Time: 8:15 Charted @: ________ Signature: [signature]
Review Time: 13:00 Signature: [signature]

Word Form # 101815 (S) 01/07 Category: Flow Sheets

Discharge Report - Multidisciplinary Progress Report		
**Day 1**<br>**9:00**	MD	Remains in observation. Continues to be SOB at rest – no real change in symptoms and cough productive of sputum. Difficulty eating because of SOB<br><br>O/E<br><br>BP 146/80, HR = 100, RR = 30, O2 sats 94% on F1O2 50%<br><br>Accessory muscle use (neck, abdomen) but alert and oriented speaking in 4-5 word sentences<br><br>Resp diffuse wheezes + decreased AE LLL<br><br>A+P/<br><br>COPD exacerbation – need to observe closely in case further respiratory support required<br><br>Continue steroids, bronchodilators, antibiotics
**Day 2**<br>**9:50**	CardioResp PT	Pt more comfortable, RR decreased to 28, speaking in full sentences without pursed-lip breathing. Reports more sputum produced after yesterday's percussion. Ausc and breath sounds bronchial LLL with course inspir crackles; generalized wheezes persist, but less dramatic. Percussed in RSL and prone/trendelenburg, spontaneous cough for small amt sticky yellow-green sputum. Taught huffing effective for large amt similar sputum. Breath sounds Increased LLL, crackles decreased and finer. Will treat again in p.m.
**Day 2**<br>**10:30**	MD	Remained SOB overnight, but was able to sleep continuing to cough. Overall feeling slightly improved this AM; difficulty eating because of SOB<br><br>Temp 38.3 HR 100, 142/80 O2 sats 94% F1O2 50%<br><br>Continues to have access m use + intercostal indrawing<br><br>Resp exam – scattered wheezes + crackles LLL post<br><br>A/ COPD exacerbation + LLL pneumonia some improvement in clinical condition, continue current therapy – prednisone, antibiotics, bronchodilators
**Day 3**<br>**9:30**	CardioResp PT	Pt reports decreased cough, decreased sputum, still very dyspneic walking bed to bathroom and back with O2 tank, OT plans to ambulate pt with walker this morning.<br><br>Ausc: breath sounds mildly decreased LLL, still coarse inspir crackles; wheezes decreased high pitched, increase with cough. Percussed ax. yesterday.<br><br>Huffed well for 1-2 ml yellowish sputum.<br><br>Crackles resolved. Plan: partner with OT to ensure progression of ambulation. Trial of PEP device for long-term secretion management
**Day 3**	COPD Nurse	Patient was seen today. Educated on pathology of COPD, and assessed inhaler technique.
**Day 3**	OT	Pt reviewed today. Patient was pleasant and agreeable with therapies. Patient indicated having some SOBOE when moving around in bed. Bed mobility: with use of bedrail; lie - sit; with bedrail; sità stand; stnd-by assist with 2ww. Bedà chair transfer: assist x1 to chair with arm rests, cueing and prompting. Toilet transfer: 16" toilet with use of grab rail x1 on L side, min assist x 1 with 2ww. Pt ambulated ~ 25 m with 2ww, assist x 1 with IV and O2. Pt became SHOBOE with transfer practices and mobility. Pl To review transfers, mobility and assess tub transfers – Signature MOTReg (Sk)

Discharge Report - Multidisciplinary Progress Report		
**Day 4**<br>**9:00**	MD	Definite improvement in respiratory status – less SOB at rest, less respiratory effort, less cough + sputum production requiring less supplemental O2 to maintain current O2 sats, eating very little<br><br>A/ improving COPD exacerbation<br><br>P/ encourage mobility – ask physio to review, ask dietitian to see re oral intake
**Day 4**<br>**10:00**	CardioResp PT	Percussion and drainage for scant sputum; Trial of PEP with 3 mm resistance – used well, produced 2-3 ml sputum. Pt will use PEP again in afternoon and evening – 5 sets x 10 breaths
**Day 5**	OT	Pt was rec'd down in OT dept. PT was pleasant and cooperative. Sit, stand from w/c: with 2ww. Toilet transfer: use of raised toilet seat and handles. Demonstrated use of transfer tub bench. Pt completed the same safely and independently. Pt agreeable to the same equipment upon d/c. Pt provided with requisition from SAIL for transfer tub bench and commode for over toilet use/ bedroom use @ night. Pl Pt will be safe for discharge – Signature MOT Reg(Sk)
**Day 5**	CPAS	Made arrangements for f/u care in the community.
**Day 5**<br>**11:10**	CardioResp PT	Pt cheerful, denies dyspnea at rest, reports "not too bad" when walking. Have loaned pt 4 wheel walker to transport O2 tank while walking. O2 @ 2.0 l/min via prongs, Sp)2 91%, supervised ambulation x 70 m – independent and safe with walker.<br><br>Discussed value of 4 wheel walker to decrease stress on hips and knees while walking; pt uncertain, but agrees he manages shopping better when he has a cart to lean on. Discussed pulmonary rehab program; pt thinks this may be worthwhile. Pt says sputum production "almost back to what I usually have" finds "it comes up easier with the PEP" PEP used for ` 2 ml yellow sputum with resistance increasing to 3.5 mm.
**Day 5**	COPD Nurse	Patient was seen today for second visit. Reviewed inhaler technique and spoke about the importance of pulmonary rehabilitation after discharge. Patient uncertain, but left door open to further discussion

Medications:

Moxifloxacin (Avelox): 400 mg po od

Spiriva (tiotroprium bromide): 18 mcg I puff od

Advair 500 diskus: 2 puffs bid

Ventolin MDI: 100 ucg: q4h prn

Prednisone: 40 mg po od

Click HERE to return to the ebook content.

Appendix 2: Mr Sim’s Care Goals

Discharge Concern	Healthcare Professional(s)
Medications: When and how to use COPD inhalers	Pharmacist,<br><br>RN from community COPD program
Shortness of breath when eating	Dietary: sufficient nutrition<br><br>Physiotherapy: techniques for breathing while eating
Follow up after discharge: who should he call and when	RN from community COPD program to describe when to call and whom<br><br>Medical resident: arrange follow up with specialist and family doctor.
Expectations regarding illness recovery (activity)	RN from community COPD program to describe when to call and whom<br><br>Medical resident: arrange follow up with specialist and family doctor.
Help at home	Home care coordinator

When you have reviewed the table, click HERE to return to the ebook content.

References (links to the papers)

1. http://www.who.int/topics/chronic_diseases/en/

2. Hall P, Weaver L. Interdisciplinary education and teamwork: a long winding road. Medical Education 2001;35: 867-875.

3. Novelli WD, Halvorson GC, Santa J. Findings from the IOM Evidence communication innovation collaborative. JAMA 2012;308(15):1531-2.

4. Parke B, Chappell N. Transactions between older people and the hospital environment: an ecologic analysis. Journal of Aging Studies 2010: 14;118-124.

5. Schmutz J, Manser T. Do team processes really have an effect on clinical performance? A systematic literature review. Br J Anaesth 2013:110(4);529-44.

6. Vedel I, Monette M, Bleand F, et al. 10 years of integrated care: backwards and forwards. The case of the province of Quebec, Canada. International Journal of Integrated Care 2011:11(7);1-11

7. Wright MC, Phillips-Bute, BG, Petrusa ER et al. Assessing teamwork in medical education and practice: relating behavioural teamwork ratings and clinical performance. Medical Teacher 2009;31:30-38.

Situational Awareness

1. http://www.cmpa-acpm.ca/cmpapd04/docs/ela/goodpracticesguide/pages/human_factors/Situational_awareness/knowing_what_is_going_on_around_you-e.html

2. Endsley, M.R. (1995). Toward a theory of situation awareness in dynamic systems. Human Factors 37(1), 32–64

Shared Mental Models

1. Carbo AR, Tess AV, Roy C, Weingart SN. Developing a high-performance team training framework for internal medicine residents: The ABC's of teamwork. J of Patient Safety 2011;7(2):72-76.

2. Mathieu J, Heffner T, Goodwin G, Salas E, Cannon-Bowers J. The Influence of Shared Mental Models on Team Process and Performance. Journal of Applied Psychology 2000;85(2):273-283.

3. Smith-Jentsch K, Mathieu J, Kraiger K. Investigating Linear and Interactive Effects of Shared Mental Models on Safety and Efficiency in a Field Setting. Journal of Applied Psychology 2005; 90(3): 523-535.

4. Westi HK, Johnsen BH, Eid J, Rasten I, Brattebo G. Teamwork skills, shared mental models, and performance in simulated trauma teams: an independent group design. Scandinavian Journal of Trauma, Resuscitation & Emergency Medicine 2010;18(47).

SBAR communication tool

1. SBAR Communication Toolkit

2. Hall P. Interprofessional teamwork: Professional cultures as barriers. Journal of Interprofessional Care 2005;supplement 1: 188-196.

Team skills learning resources:

1. http://www.cihc.ca/

2. http://nexusipe.org

Acknowledgements:

UNIVERSITY OF SASKATCHEWAN

Saskatoon Health Region

Saskatchewan Academic Health Sciences Network

THE GWENNA MOSS CENTRE
FOR TEACHING EFFECTIVENESS
